# Supplementary material for: Assessing health governance across countries: a scoping review protocol on indices and assessment tools applied globally
Source: BMJ Open. 2022 Jul 14;12(7):e063866. doi: 10.1136/bmjopen-2022-063866 (PMC9295668; doi:10.1136/bmjopen-2022-063866)
Supplement: Supplementary data [file bmjopen-2022-063866supp001.pdf]

Appendix A to Appendix D

Appendix A. KEY CONCEPTS

Governance

Governance has been studied in various dimensions, such as socio-legal studies, political science, economics, and development studies. In a broader term, governance is described as a series of collective actions and decision-making procedures with diverse actors and organisations without formal control mechanisms. Governance does not depend on authority and coercion; it is achieved through negotiation, communication, and hegemonic influence.(1) It does not only concern the government, and it emphasises governing with and through networks between public, private and voluntary sectors.(2) The United Nations Development Programme (UNDP) refers to good governance broadly as the principles of legitimacy and voice, direction, performance, accountability, and fairness.(3) Definition of governance World Health Organization (WHO) focuses on effective oversight, coalition-building, the provision of regulations and incentives, attention to system-design and accountability.(4) Concerning health, Baez-Camargo and Jacobs define governance as "processes through which health systems manage human resources, acquire and distribute medicines and technologies, generate and disseminate information, and provide means to finance the provision of health services to the population." (5)

Given the inclusiveness of "governance", this scoping review does not aim to define a concrete *priori* concept of governance. Rather, it tried extensively potential search terms according to the existing analysis of governance dimensions (**Table A**) through a rapid review of reviews (systematic review, scoping review or systematic-like reviews).[1] Three reviews were eligible as they synthesised the literature surrounding the concept of "governance". The first column lists the elements, dimensions or functions (referred to as "dimensions) of "governance" identified by these three reviews or their search terms for "governance". To further identify the more recognised key dimensions of "governance", this column only shows the dimensions or search terms overlapped by at least two reviews. Terms with similar meanings are classified in one group, such as participation and involvement. The second to fourth column shows the original expressions of the classified dimensions or search terms in the corresponding reviews.

Based on Table A, we develop the search terms for the concept "governance" in our search strategy. The search terms cover all the dimensions or search terms for "governance" overlapped in the included review listed in Table A.

Besides, we also referred to search strategies of existing systematic reviews or scoping reviews on health governance[2].(6–12) Apart from "governance" itself and the dimensions listed above, we add

[1] We used the search strings "governance AND (concepts OR concept OR definition* OR define OR defining OR meaning*)" in Web of Science, Ovid (Embase <1974 to 2022 March 11> and Medline and Epub Ahead of Print, In-Process, In-Data-Review & Other Non-Indexed Citations, Daily and Versions <1946 to March 11, 2022>), Cochrane, and Google Scholar. The search field was "Title" in bibliographic databases, the time frame was 2000-2021 and the document type was review article. Without removing duplicates, the searching results are 360, 178 and 5220 respectively in Web of Science, Ovid and Cochrane. In order to find additional literature to the bibliographic databases, there is no search field in Google Scholar and the top 100 items by relevance were screened. The search date was 14 March 2022. In addition, in Barbazza et al. (2014), broad dimensions include some fundamental values and outcomes that could be independent from the concept of governance, so we only list the functional dimensions according to this article in Table A.

[2] We used the search strings "health NEAR/5 governance' AND ('systematic review' OR 'scoping review')" in Web of Science and Ovid (Embase <1974 to 2022 March 11> and Medline and Epub Ahead of Print, In-Process, In-Data-Review & Other Non-Indexed Citations, Daily and Versions <1946 to March 11, 2022>). The search field was "Title" and the time frame was 2000-2021. Without removing duplicates, the searching results are 15 and 10 respectively in Web of Science and Ovid. The search date was 14 March 2022. The inclusion criteria are: (1) using governance as a concept to search; (2) the search terms in the governance concept are more than "governance" itself; (3) the topic is health related governance, not data or other governance or other governance

another term, “leadership”, for two reasons. First, it has been widely used as a search term for the concept of “governance”. We did another review of reviews focusing on search terms used in systematic or scoping reviews on health governance and found that three out of seven eligible reviews used “leadership” as a search term for the concept of “governance”.(6–8) Except for “governance” per se, only “accountability” appears in these reviews more than “leadership” does (four times). Second, leadership entails “the capacity of the system to initiate, implement and monitor a regulatory system” and “the ability to organise and manage the alignment of all relevant actors and actions engaged in processes pertaining to this”.(13) Thus, it is often linked to governance, as explicated in governance literature (13,14) and potentially eligible literature in this scoping review like Leadership and Governance for Health Indicators.(15)

Table A. Summary of reviews regarding governance dimensions

Information of eligible reviews / *Classified dimensions or search terms for “governance”*	*Carlson et al., “Defining the functions of public health governance.” American Journal of Public Health (2015).(14)*	*Barbazza and Tello, “A review of health governance: definitions, dimensions and tools to govern.” Health Policy (2014). (13)*	*Ruhanen et al. “Governance: a review and synthesis of the literature.” Tourism Review (2010).(16)*
Accountability	-	Accountability	Accountability
Stewardship	Resource stewardship	Stewardship	-
Transparency	-	Transparency	Transparency
Policy development/formulation	Policy development	Formulating policy/strategic direction	-
Strategic vision/direction	-	Formulating policy/strategic direction	Strategic vision
Partner/partnership	Partner engagement	Partnerships	-
Participation/involvement	-	Participation and consensus	Involvement
Consensus	-	Participation and consensus	Consensus

Global Health Governance

While titled “health governance across countries”, this scoping review is embedded in the context of global health governance. Although existing literature has centred on governance in health systems and health development only in recent decades, there has been an increasing interest in discussing the relationship between governance and global health. Despite the extensive scholarly debates on the definition of global health, it is still elusive to reach a consensus around a precise definition. In 2009, Koplan and colleagues argued for “a common definition of global health”, which emphasises transnational health, and embraces different disciplines and interdisciplinary collaboration.(17) Scholars delineate global health by focusing on multiple dimensions, such as education, governance and security. Many also view global health as a mode of governance across borders.(18) The term global health governance (GHG) is widely used in scholarly work, but few researchers agree on how the term should be applied. Lee and Kamradt-Scott point out that the GHG peer-reviewed literature varies substantially on what kinds of and to what extent institutions should engage in GHG. In addition, the goal and function of GHG are also not clear.(19) For instance, Kickbusch and Szabo refer GHG mainly to institutions and governance processes that are directly linked to health, such as the WHO.(20) Another highly cited definition proposed by David P. Fidler takes a more inclusive approach. He defines GHG as “the use of formal and informal institutions, rules, and processes by states,

linking to health; (4) systematic review or scoping review. Seven literature is eligible as a result of this review of reviews.

intergovernmental organisations, and non-state actors to deal with challenges to health that require cross-border collective action to address effectively.” (21)

Appendix B. PRELIMINARY SEARCHES

The methods for searching related published/ongoing reviews were: (1) directly searching using strings “'health governance' AND ('index' OR 'indicator' OR assess) AND ('systematic review' OR 'scoping review')"; (2) forward snowballing for the reviews including our potential eligible literature such as GHSI and IHR core capacity, using strings "('global health security index' OR 'GHSI') AND ('systematic review' OR 'scoping review')" and "('international health regulations' OR 'IHR') AND ('systematic review' OR 'scoping review') AND ('core capacity' OR 'score')". There was no filter or limit, and the search dates were 20 November 2021 and 22 March 2022.

Appendix C. FULL SEARCH STRATEGY

Web of science Core Collection

Search date: 03 April 2022

No.	*Query*	*Results*
#1	TS=(“health” OR “healths” OR "healthcare*")	2,899,246
#2	TS=("governance*" OR "leadership*" OR "accountabilit*" OR "stewardship*" OR (("policy" OR "policies") NEAR/10 (“develop” OR “developing” OR “develops” OR “developed” OR “development” OR “developments” OR “formulat*”)) OR ("strateg*" NEAR/10 (“vision” OR “visions” OR “direction” OR “directions”)) OR "partner*" OR "transparen*" OR "participation*" OR "involvement*" OR "consensus*")	2,178,485
#3	TS=("evaluate" OR "evaluated" OR "evaluates" OR "evaluating" OR "evaluation*" OR "monitor" OR "monitors" OR "monitored" OR "monitoring" OR "measure " OR "measures" OR "measured" OR "measuring" OR "measurement*" OR "assess" OR "assesses" OR "assessed" OR "assessing" OR "assessment*")	15,885,119
#4	TS=("indicator*" OR "score" OR "scores" OR "scored" OR "scoring" OR "index" OR "indexs" OR "indexes" OR "indexed" OR "indices" OR "indexing")	3,259,711
#5	TS=("globe" OR "global" OR "globally" OR "international" OR "internationally" OR "world" OR "worldwide" OR "worldwidely" OR "multi country " OR "multi countries”)	3,745,205
#6	#1 AND #2 AND #3 AND #4 AND #5 AND DOP=(2000-01-01/2021-12-31)	8,046
#7	#7 NOT DT=(Review OR Editorial Material OR Meeting Abstract OR Letter)	6,844

PubMed

Search date: 03 April 2022

No	*Query*	*Results*
#1	"health"[MeSH Terms] OR "health"[Title/Abstract] OR "healths"[Title/Abstract] OR "healthcare*"[Title/Abstract]	2,617,637
#2	"leadership"[MeSH Terms] OR "public policy"[MeSH Terms] OR "consensus"[MeSH Terms] OR "social participation"[MeSH Terms] OR "community participation"[MeSH Terms] OR "stakeholder participation"[MeSH Terms] OR "governance*"[Title/Abstract] OR "leadership*"[Title/Abstract] OR "accountabilit*"[Title/Abstract] OR "stewardship*"[Title/Abstract] OR (("policy"[Title/Abstract] OR "policies"[Title/Abstract]) AND (("develop"[Title/Abstract] OR "developing"[Title/Abstract] OR "develops"[Title/Abstract] OR "developed"[Title/Abstract] OR "development"[Title/Abstract] OR "developments"[Title/Abstract]) OR "formulat*"[Title/Abstract])) OR ("strateg*"[Title/Abstract] AND (“vision”[Title/Abstract] OR “visions”[Title/Abstract] OR “direction”[Title/Abstract] OR “directions”[Title/Abstract])) OR "partner*"[Title/Abstract] OR "transparen*"[Title/Abstract] OR "participation*"[Title/Abstract] OR	1,434,193

	"involvement*"[Title/Abstract] OR "consensus*"[Title/Abstract]	
#3	"evaluate"[Title/Abstract] OR "evaluated"[Title/Abstract] OR "evaluates"[Title/Abstract] OR "evaluating"[Title/Abstract] OR "evaluation*"[Title/Abstract] OR "monitor"[Title/Abstract] OR "monitors"[Title/Abstract] OR "monitored"[Title/Abstract] OR "monitoring"[Title/Abstract] OR "measure"[Title/Abstract] OR "measures"[Title/Abstract] OR "measured"[Title/Abstract] OR "measuring"[Title/Abstract] OR "measurement*"[Title/Abstract] OR "assess"[Title/Abstract] OR "assesses"[Title/Abstract] OR "assessed"[Title/Abstract] OR "assessing"[Title/Abstract] OR "assessment*"[Title/Abstract]	9,147,656
#4	"indicator*"[Title/Abstract] OR "score"[Title/Abstract] OR "scores"[Title/Abstract] OR "scored"[Title/Abstract] OR "scoring"[Title/Abstract] OR "index"[Title/Abstract] OR "indexs"[Title/Abstract] OR "indexes"[Title/Abstract] OR "indexed"[Title/Abstract] OR "indices"[Title/Abstract] OR "indexing"[Title/Abstract]	2,311,393
#5	"globe"[Title/Abstract] OR "global"[Title/Abstract] OR "globally"[Title/Abstract] OR "international"[Title/Abstract] OR "internationally"[Title/Abstract] OR "world"[Title/Abstract] OR "worldwide"[Title/Abstract] OR "worldwidely"[Title/Abstract] OR "multi country"[Title/Abstract] OR "multi countries"[Title/Abstract]	1,490,795
#6	#1 AND #2 AND #3 AND #4 AND #5	7,928
#7	#1 AND #2 AND #3 AND #4 AND #5 AND ("2000/01/01"[Date - Publication] : "2021/12/31"[Date - Publication])	7,407
#8	(#7) NOT (Comment[Publication Type] OR Editorial[Publication Type] OR Letter[Publication Type] OR Review[Publication Type])	6,270

Embase

Search date: 03 April 2022

No.	*Query*	*Results*
#1	'health'/exp OR 'public health'/exp OR 'health care'/exp OR health:ab,ti,kw OR healths:ab,ti,kw OR 'healthcare*':ab,ti,kw	11,573,270
#2	'leadership'/exp OR 'policy'/exp OR 'consensus'/exp OR 'governance'/exp OR 'accountability'/exp OR 'strategy'/exp OR 'partner'/exp OR 'partnership'/exp OR 'participation'/exp OR 'involvement'/exp OR 'transparency'/exp OR 'governance*':ab,ti,kw OR 'leadership*':ab,ti,kw OR 'accountabilit*':ab,ti,kw OR 'stewardship*':ab,ti,kw OR (('policy' OR 'policies') NEAR/10 ('develop' OR 'developing' OR 'develops' OR 'developed' OR 'development' OR 'developments' OR 'formulat*')):ab,ti,kw OR ('strateg*' NEAR/10 ('vision' OR 'visions' OR 'direction' OR 'directions')):ab,ti,kw OR 'partner*':ab,ti,kw OR 'transparen*':ab,ti,kw OR 'participation*':ab,ti,kw OR 'involvement*':ab,ti,kw OR 'consensus*':ab,ti,kw	1,866,006
#3	'monitor'/de OR 'measurement'/de OR 'assessment'/exp OR evaluate:ab,ti,kw OR evaluated:ab,ti,kw OR evaluates:ab,ti,kw OR evaluating:ab,ti,kw OR evaluation*:ab,ti,kw OR monitor:ab,ti,kw OR monitors:ab,ti,kw OR monitored:ab,ti,kw OR monitoring:ab,ti,kw OR measure:ab,ti,kw OR measures:ab,ti,kw OR measured:ab,ti,kw OR measuring:ab,ti,kw OR measurement*:ab,ti,kw OR assess:ab,ti,kw OR assesses:ab,ti,kw OR assessed:ab,ti,kw OR assessing:ab,ti,kw OR assessment*:ab,ti,kw	12,334,482
#4	'indicator'/de OR 'score'/exp OR 'index'/exp OR indicator*:ab,ti,kw OR score:ab,ti,kw OR scores:ab,ti,kw OR scored:ab,ti,kw OR scoring:ab,ti,kw OR index:ab,ti,kw OR indexs:ab,ti,kw OR indexes:ab,ti,kw OR indexed:ab,ti,kw OR indices:ab,ti,kw OR indexing:ab,ti,kw	3,358,824
#5	'global'/exp OR 'world'/exp OR globe:ab,ti,kw OR global:ab,ti,kw OR globally:ab,ti,kw OR international:ab,ti,kw OR internationally:ab,ti,kw OR world:ab,ti,kw OR worldwide:ab,ti,kw OR worldwidely:ab,ti,kw OR 'multi country':ab,ti,kw OR 'multi countries':ab,ti,kw	2,099,845
#6	#1 AND #2 AND #3 AND #4 AND #5	19,014
#7	#1 AND #2 AND #3 AND #4 AND #5 AND [2000-2021]/py	18,199
#8	#7 NOT (editorial:it OR letter:it OR review:it OR 'conference abstract':it)	9,416

Google

Search date: 03 April 2022

Customed range: 2000-2021

No.	*Query (Google limits queries to 32 words)*	*Results*
1	health AND (governance OR leadership OR accountability OR stewardship) AND (evaluate OR monitor OR measure OR assess) AND (index OR indicator OR score) AND (global OR international OR world OR multi-country)	151
2	health AND (transparency OR policy OR strategy) AND (evaluate OR monitor OR measure OR assess) AND (index OR indicator OR score) AND (global OR international OR world OR multi-country)	178
3	health AND (partnership OR participation OR involvement OR consensus) AND (evaluate OR monitor OR measure OR assess) AND (index OR indicator OR score) AND (global OR international OR world OR multi-country)	157

Appendix D. ELIGIBILITY CRITERIA IN DETAIL

	Inclusion criteria	*Exclusion criteria*
Subjects	Indices or assessment tools on human, animal, and/or environmental health governance with measurable indicators:<br>1.1 General health governance;<br>1.2 Governance of health system;<br>1.3 Governance of public health risks such as public health emergencies, epidemics and pandemics;<br>1.4 Governance on specific health issues such as influenza, reproductive health.	Assessment/conceptual frameworks, or narrative assessment without measurement; on topics irrelevant to health<br>1.1 Not being an index or assessment tool;<br>1.2 Irrelevant to our topics<br>1.2.1 Not governance-related;<br>1.2.2 Not health-related;<br>1.2.3 Health outcome-based;<br>1.2.4 Both non-governance- and non-health-related
Objectives	2 Describing the indices or assessment tools (incl. indicators or scoring results)	2 Only criticising, mentioning, and analysing the indices or assessment tools while not aiming to yield assessment results for health governance
Coverage	3 Can be applied in multi-countries at the global level (although the assessed subject might be sub-national/national entities)	3.1 Geographical coverage: Only applied/can be applied within one country or one region;<br>3.2 Entity coverage: Assessing one type of specific organisations or individuals (e.g., hospital or enterprise)<br>3.3 Case study: Appearing as a case study without further generalisation
Type of sources	4 Reports, documents, peer-reviewed publications, websites	4 Wrong publication types: commentaries, reviews, blogs, protocols

REFERENCES

1. Chhotray V, Stoker G. Governance: From Theory to Practice. In: Chhotray V, Stoker G, editors. Governance Theory and Practice: A Cross-Disciplinary Approach [Internet]. London: Palgrave Macmillan UK; 2009 [cited 2022 Mar 23]. p. 214–47. Available from: https://doi.org/10.1057/9780230583344_10

2. Rhodes RAW. Understanding Governance: Ten Years On. Organ Stud. 2007 Aug;28(8):1243–64.

3. Nations DPU. Governance for sustainable human development: A UNDP policy document. UNDP; 1997.

4. World Health Organization. Everybody's business–strengthening health systems to improve health outcomes: WHO's framework for action. 2007;

5. Baez Camargo C, Jacobs E. A framework to assess governance of health systems in low income countries. 2011;

6. Hastings S, Armitage G, Mallinson S, Jackson K, Suter E. Exploring the relationship between governance mechanisms in healthcare and health workforce outcomes: a systematic review. BMC Health Serv Res. 2014 Oct 4;14.

7. Pyone T, Smith H, van den Broek N. Frameworks to assess health systems governance: a systematic review. Health Policy Plan. 2017 Jun 1;32(5):710–22.

8. Abd Rahim A, Manaf R, Juni M, Ibrahim N. Health System Governance for the Integration of Mental Health Services into Primary Health Care in the Sub-Saharan Africa and South Asia Region: A Systematic Review. Inq- J Health CARE Organ Provis Financ. 2021 Jul;58.

9. Mohammed K, Ahmed B. The governance of basic health coverage: A systematic review. Ethiop J Health Dev [Internet]. 2020 Oct 22 [cited 2021 Nov 20];34(3). Available from: https://www.ajol.info/index.php/ejhd/article/view/201342

10. Naher N, Hoque R, Hassan M, Balabanova D, Adams A, Ahmed S. The influence of corruption and governance in the delivery of frontline health care services in the public sector: a scoping review of current and future prospects in low and middle-income countries of south and south-east Asia. BMC PUBLIC Health. 2020 Jun 8;20(1).

11. Ayanore M, Amuna N, Aviisah M, Awolu A, Kipo-Sunyehzi D, Mogre V, et al. Towards Resilient Health Systems in Sub-Saharan Africa: A Systematic Review of the English Language Literature on Health Workforce, Surveillance, and Health Governance Issues for Health Systems Strengthening. Ann Glob Health. 2019;85(1).

12. Tabrizi J.S., Pourasghar F., Nikjoo R.G. Primary health care governance pillars and components in developed countries, a systematic review. Int J Pharm Technol. 2016;8(4):24045–59.

13. Barbazza E, Tello JE. A review of health governance: Definitions, dimensions and tools to govern. Health Policy. 2014 May 1;116(1):1–11.

14. Carlson V, Chilton MJ, Corso LC, Beitsch LM. Defining the Functions of Public Health Governance. Am J Public Health. 2015 Apr;105(S2):S159–66.

15. Abt Associates. Leadership and Governance for Health Indicators | HFG [Internet]. The Health Finance and Governance (HFG) Project. [cited 2021 Nov 19]. Available from: https://www.hfgproject.org/health-governance-indicators/

16. Ruhanen L, Scott N, Ritchie B, Tkaczynski A. Governance: a review and synthesis of the literature. Pechlaner H, Raich F, Beritelli P, editors. Tour Rev. 2010 Jan 1;65(4):4–16.

17. Koplan JP, Bond TC, Merson MH, Reddy KS, Rodriguez MH, Sewankambo NK, et al. Towards a common definition of global health. The Lancet. 2009 Jun 6;373(9679):1993–5.

18. Salm M, Ali M, Minihane M, Conrad P. Defining global health: findings from a systematic review and thematic analysis of the literature. BMJ Glob Health. 2021;6:1–14.

19. Lee K, Kamradt-Scott A. The multiple meanings of global health governance: a call for conceptual clarity. Glob Health. 2014;10(1):28.

20. Kickbusch I, Szabo MMC. A new governance space for health. Glob Health Action. 2014 Feb 13;7(s2):23507.

21. Kickbusch I. Global Health Governance Challenges 2016 – Are We Ready? Int J Health Policy Manag. 2016 Feb 29;5(6):349–53.
