# Supplementary material for: Assessing health governance across countries: a scoping review protocol on indices and assessment tools applied globally
Source: BMJ Open. 2022 Jul 14;12(7):e063866. doi: 10.1136/bmjopen-2022-063866 (PMC9295668; doi:10.1136/bmjopen-2022-063866)
Supplement: Author's manuscript [file bmjopen-2022-063866.draft_revisions.pdf]

BMJ Open

BMJ Open is committed to open peer review. As part of this commitment we make the peer review history of every article we publish publicly available.

When an article is published we post the peer reviewers' comments and the authors' responses online. We also post the versions of the paper that were used during peer review. These are the versions that the peer review comments apply to.

The versions of the paper that follow are the versions that were submitted during the peer review process. They are not the versions of record or the final published versions. They should not be cited or distributed as the published version of this manuscript.

BMJ Open is an open access journal and the full, final, typeset and author-corrected version of record of the manuscript is available on our site with no access controls, subscription charges or pay-per-view fees (http://bmjopen.bmj.com).

If you have any questions on BMJ Open's open peer review process please email info.bmjopen@bmj.com

BMJ Open

# Assessing Health Governance Across Countries: A Scoping Review Protocol on Indexes and Assessment Tools Applied Globally

| | |
|---|---|
| Journal: | *BMJ Open* |
| Manuscript ID | bmjopen-2022-063866 |
| Article Type: | Protocol |
| Date Submitted by the Author: | 14-Apr-2022 |
| Complete List of Authors: | Huang, Aidan; Tsinghua University, Vanke School of Public Health; Tsinghua University, Institute for Healthy China<br>Lin, Yuling; University of Geneva Global Studies Institute<br>Zhang, Liyuan; University of Cambridge<br>Dong, Jingwen; Shanghai Jiao Tong University School of Public Health<br>Tang, Kun; Tsinghua University, Vanke School of Public Health; Tsinghua University, Institute for Healthy China<br>He, Qiwei; Tsinghua University, Vanke School of Public Health; Tsinghua University, Institute for Healthy China |
| Keywords: | Health policy < HEALTH SERVICES ADMINISTRATION & MANAGEMENT, PUBLIC HEALTH, Public health < INFECTIOUS DISEASES |
| | |

SCHOLARONE™ Manuscripts

# Assessing Health Governance Across Countries: A Scoping Review Protocol on Indexes and Assessment Tools Applied Globally

**Aidan Huang[1], Yuling Lin[2], Liyuan Zhang[3], Jingwen Dong[4], Kun Tang[5], Qiwei He[6]**

1. Vanke School of Public Health, Tsinghua University, Beijing, China; Institute for Healthy China, Tsinghua University, Beijing, China. Email: adhuang@mail.tsinghua.edu.cn
2. Global Studies Institute, University of Geneva, 1205 Geneva, Switzerland. Email: yuling.lin@etu.unige.ch
3. University of Cambridge, Cambridge CB2 1TN, United Kingdom. Email: lz449@cam.ac.uk
4. School of Public Health, Shanghai Jiao Tong University, Shanghai, China. Email: ilovemath@sjtu.edu.cn
5. Vanke School of Public Health, Tsinghua University, Beijing, China; Institute for Healthy China, Tsinghua University, Beijing, China. Email: tangk@mail.tsinghua.edu.cn.

6. Vanke School of Public Health, Tsinghua University, Beijing, China; Institute for Healthy China, Tsinghua University, Beijing, China. Email: heqiwei@mail.tsinghua.edu.cn

**Correspondence to Dr Kun Tang**; tangk@mail.tsinghua.edu.cn. Postal address: Vanke School of Public Health, Tsinghua University, No. 30 Shuangqing Road, Beijing, 100084, China. Telephone: +86-13671129425.

Word count: 2563

For peer review only

# Abstract

**Introduction:** The failure of global health efforts might be attributed to the lack of a solid governance framework under international anarchy. To benchmark equitable and solidary global health governance, it becomes necessary to reflect on the current state of indexes or assessment tools evaluating health governance across countries. This scoping review aims to (1) review the existing multi-country indexes or assessment tools applied globally with measurable indicators assessing health governance; (2) summarise their differences and commons in health topics, purposes, contents, methods, and operation; (3) identify the lessons learned through analysis of their strengths and gaps; and (4) evaluate the feasibility and necessity to establish a new index or consensus framework for assessing global health governance.

**Methods and analysis:** This scoping review protocol follows Arksey and O'Malley's methodological framework, the Joanna Briggs Institute (JBI) guidelines and the Preferred Reporting Items for Systematic Reviews and Meta-analyses (PRISMA) methodology for scoping reviews. Key information sources will be bibliographic databases (PubMed, Embase, and Web of Science Core Collection), grey literature and citation tracking. The time frame will be 1 January 2000 to 31 December 2021. Only indexes or assessment tools that are globally applicable and provide measurable indicators of health governance will be eligible.

**Ethics and dissemination:** This scoping review does not require ethics approval. Dissemination will include a peer-review article, policy briefs and conference presentations. This protocol has been registered in the Open Science Framework (osf.io/y93mj).


**Keywords:** health governance; index; assessment tool; global health; scoping review protocol

# Article Summary

- This scoping review will be a prior assessment in establishing a new index or consensus framework for assessing global health governance for the post-COVID-19 era.
- This scoping review will differ from the existing reviews by incorporating governance for a wide range of health objectives and broadening geographic coverage with a global lens.
- The literature to be reviewed will include research articles and indexes or assessment tools used by organisations, with theoretical and practical implications for assessing health governance.
- This protocol has been refined by pilot tests in searching and study selection and consulting with multiple librarians.
- With the topic being broad and interdisciplinary, the precision of the search strategy might be constrained.

For peer review only

# INTRODUCTION

## Rationale

The health governance of countries shapes global health governance. In a broad sense, governance is described as a series of collective actions and decision-making procedures with diverse actors and organisations without formal control mechanisms.(1) Governance emphasises governing with and through networks between public, private and voluntary sectors.(2) It is one of the blocks in the widely-used health systems framework formulated by the World Health Organization (WHO).(3) Given the globalised health issues, health governance in each sovereignty has been closely linked. From the pandemic of SARS to the COVID-19, repeating global health crises have alerted the need for global health solidarity efforts.(4) The failure of such efforts might be attributed to the lack of a solid governance framework under international anarchy(5–7), although United Nations' 2030 Sustainable Development Goals (SDGs) have set up goals to promote global health outcomes.

Indeed, existing indexes or assessment tools in global health tend to focus on health outcomes instead of the governance elements attributed to these outcomes (see Appendix A in the supplemental material). Even within health governance, multiple parallel overlapping frameworks, assessment tools and indexes for theoretical or practical purposes have created complexities. Besides, 85 per cent of global health organisations have their headquarters in Europe or North America; more than 80 per cent of the global health leaders come from high-income countries. (8) Therefore, most global health indexes or assessment tools and indicators have been produced from high-income countries' perspectives, failing to reflect the other populations. Due to economic constraints and low logistic capacity, health statistics in developing countries are with varying standards and difficult-to-assess accuracy.(9) Thus, global health indicators' validity, utility, and representativeness in developing countries are questionable.(10)

The underlying standpoint of this scoping review is that, with the deeply-rooted notions of sovereignty under "international anarchy", global health governance has to be anchored around the health governance of countries. A starting point might be a consensus framework or a new, integrated index on health governance across countries globally. Thus, scoping the existing indexes and assessment tools will lay out a practical basis for developing an index or consensus framework to benchmark equitable, solidary global health governance.

## Objectives

This scoping review aims to (1) review the existing multi-country indexes or assessment tools applied globally with measurable indicators assessing health governance; (2) summarise their differences and commons in health topics, purposes, contents, methods, and operation; (3) identify the lessons learned through analysis of their strengths and gaps; and (4) assess the feasibility and necessity to establish a new index or consensus framework for assessing global health governance.

As global health governance is an emerging, multidisciplinary field, a scoping review is a more appropriate tool to "assess and understand the extent of the knowledge and identify, map, report, or discuss the characteristics or concepts".(11) By contrast, systematic reviews aiming to "answer a clinically meaningful question or provide evidence to inform practice"(12), or


For peer review only - http://bmjopen.bmj.com/site/about/guidelines.xhtml

literature reviews with less systematic, transparent and reproducible methods will not meet the objectives above.

## Eligible literature

Index and assessment tools are both tools for evaluation with measurable indicators. In practice, "index" is often an external evaluation tool resulting in scores or rankings, while "assessment tool" often refers to guidance or checklist for benchmarked standards (it might be called "self-assessment tool" in some cases).

Only indexes or assessment tools that are globally applicable and provide measurable indicators of health governance will be eligible. International institutions, universities and think tanks might have established the majority of the potentially eligible literature, such as the Global Health Security Index (GHSI) by Threat Initiative (NTI), the Johns Hopkins Center for Health Security and Economist Impact, International Health Regulations (IHR) Monitoring & Evaluation Framework by the WHO, and the Health System Assessment Approach by the United States Agency for International Development (USAID). Some other potentially eligible literature can also be found in bibliographic databases, such as the "health development governance index".(10) In the health sector, the authors could only find indexes or assessment tools to evaluate national or sub-national governance, although the assessment results might be comparable across countries under international coordination. Therefore, the authors posit that the assessment of transnational, multinational, international or global governance might be rare. However, the authors will include the latter pieces of literature if there are any.

This scoping review excludes assessment frameworks without measurable indicators for the following reasons. First, there have been scoping reviews, systematic reviews or review protocols covering health governance frameworks in the health system(13–15), health emergencies or health security(16) or both(17,18), while few of them pragmatically concentrate on indexes or assessment tools. Second, most health governance frameworks have not been applied in practice, and there is a lack of real-world evidence to validate the efficacy of these frameworks. Pyone, Smith and van den Broek found that within 16 frameworks for assessing governance in the health system, only five were applied in empirical research.(15) Mikkelsen-Lopez and her colleagues also point out that the lack of empirical work might result from unrealistic indicators and overly complicated framework design.(19)

This scoping review also excludes indexes or assessment tools designed to be applied in a particular country or region. Some reviews have included indexes or assessment tools applied in regions like Europe as part of eligible literature(20–22). Moreover, considering the objectives of this scoping review, including indexes or assessment tools applied in particular countries or regions will weaken the global generalisability. Moreover, since the concept of "governance" in this scoping review involves diverse actors and organisations, governance of only one type of organisation (e.g., hospital or enterprise) does not fit this research's scope.

## Related published/ongoing reviews

The authors did not identify any published or ongoing systematic reviews or scoping reviews on the topic through a preliminary search in Google Scholar, PROSPERO, JBI Evidence Synthesis, Figshare, Open Science Framework, and Research Gate (see Appendix B in the supplemental material for the methods of the preliminary search). Some eligible indexes or assessment tools included in similar reviews(22) will be included and analysed in this scoping review, although their objectives and analytical methods differ from those of this review.


For peer review only - http://bmjopen.bmj.com/site/about/guidelines.xhtml

Specifically, this scoping review will differ from the existing reviews by (1) incorporating governance for a wide range of health objectives, such as health system strengthening (including universal health coverage) and health security (including public health emergency preparedness); (2) broadening the geographic coverage with a global lens; (3) focusing on indexes or assessment tools in practice to inform decision-making for future assessment of global health governance.

# METHODS

This scoping review protocol follows Arksey and O'Malley's methodological framework(23), the Joanna Briggs Institute (JBI) guidelines(24) and the Preferred Reporting Items for Systematic Reviews and Meta-analyses (PRISMA) methodology for scoping reviews(11,25). The reviewers also refer to systematic review methods (e.g., search strategy and reporting) that might assist the transparency and rigorousness of this scoping review.(26–30)

This protocol has been registered in the Open Science Framework (osf.io/y93mj). The searches were conducted in each proposed information source on 3 April 2022. The following research and writing will start in June 2022 and last 2-3 months. The final scoping review will report important protocol amendments and their rationales.

## Research questions

Following the objectives of this scoping review, the following research questions will guide the study:

• What indexes or assessment tools are designed to be applied globally with measurable indicators assessing health governance across multiple countries?

• What are their differences and commons in health topics, purposes, contents, methods and operation approaches?

• Which are the strengths and limitations that can learn from the existing literature to inform the future global health governance index or consensus framework development?

## Identifying relevant studies

### Electronic Searches

The search strategy will locate both publications in bibliographic databases and grey literature and adapt for each included information source. Given that only the term "health governance" started to appear in the published literature around 2000, the search will be filtered by the publication dates between 1 January 2000 and 31 December 2021. The Peer Review of Electronic Search Strategies (PRESS) checklist has been used for the proposed full search strategy.(30)

Our search terms come from the following sources: (1) concepts related to research questions; (2) MeSH and Emtree databases; (3) completed and ongoing related systematic reviews and scoping reviews. Using Table 1, the authors join all terms within each concept with OR and join each concept together using AND.



For peer review only - http://bmjopen.bmj.com/site/about/guidelines.xhtml

**Table 1.** Search terms

| ***Key concepts*** | ***Health*** | ***Governance*** | ***Assess*** | ***Measuring tools*** | ***Global*** |
|---|---|---|---|---|---|
| ***Search terms*** | health | 1. governance<br>2. leadership<br>3. accountability<br>4. stewardship<br>5. transparency<br>6. policy development/formulation<br>7. strategic vision/direction<br>8. partnership<br>9. participation<br>10. involvement<br>11. consensus | 1. evaluate<br>2. monitor<br>3. measure<br>4. assess | 1. indicator<br>2. score<br>3. index | 1. global<br>2. international<br>3. world<br>4. multi-country |

The authors will search the following bibliographic databases: PubMed, Embase and Web of Science Core Collection. Appendix C in the supplemental material presents a full search strategy for each electronic database.

Given that some indexes or assessment tools might not be commercially or academically published, grey literature will be an essential source of information in this review. Google will be searched using a de-customised mode. Other search tools will include WHO Institutional Repository for Information Sharing (IRIS). In addition, experts in global health will be consulted to explore additional literature sources.

### Citation Tracking

As the meaning of "governance" in this review might not be apparent in the existing indexes or assessment tools, citation tracking will be used to identify relevant articles. One approach is backwards snowballing (reference searching) through reviews or literature citing a potentially eligible index or assessment tool. For example, the scoping review by Chiossi, Tsolova, and Ciotti might have included some potential eligible literature for this review.(22) Another approach is forward snowballing (cited by searching) through eligible literature. Citation tracking in the related field of literature can support us in finding additional indexes and assessment tools.

## Selection of eligible studies

The literature that meets all the inclusion criteria will be included, while literature that meets any one of the exclusion criteria will be excluded. **Table 2** presents the eligibility criteria, following the SOCT (Subjects, Objectives, Coverage, Type of sources) framework developed by the authors. Appendix D in the supplemental material presents detailed eligibility criteria to assist the reviewers' decision in study selection.

All literature searched through bibliographic databases will be uploaded to Covidence, which will identify and remove duplications according to the titles and abstracts. Based on the eligibility criteria, two independent reviewers will screen the titles and abstracts (and full texts if no clues are helping to judge the eligibility) and then assess the full texts in detail to select the literature. However, for Google and WHO IRIS, another two reviewers will de-customise the searching, export the results for each search string to Excel, screen the titles and



For peer review only - http://bmjopen.bmj.com/site/about/guidelines.xhtml

abstracts, summaries, or introductions if applicable, and then assess the full texts in detail separately. Literature obtained from citation tracking will be selected after the selection process of literature obtained from electronic searches.

**Table 2**. Eligibility criteria: SOCT framework

| | *Inclusion criteria* | *Exclusion criteria* |
|---|---|---|
| *Subjects* | Indexes or assessment tools on health governance with measurable indicators | Assessment frameworks or conceptual frameworks, or narrative assessments without measurement; on topics irrelevant to health |
| *Objectives* | Describing the indexes or assessment tools (including. indicators or scoring system) | Only criticising, mentioning, analysing the indexes or assessment tools while not aiming to yield assessment results for health governance |
| *Coverage* | Can be applied in multiple countries at the global level | Applied or can only be applied within one country, one region or one type of specific organisations or individuals (e.g., hospital, enterprise); only appearing as a case study without further generalisation |
| *Type of sources* | Reports, documents, peer-reviewed publications, websites | Commentaries, editorials, reviews, blogs, letters, conference abstracts, protocols |

A pilot test with randomly selected 50 samples will be conducted. The reviewers will meet to discuss discrepancies and modify the eligibility criteria and elaboration document. The screening will only start when 75% agreement is achieved.(24)

The reasons for any exclusion following the full-text review will be recorded. The reviewers will resolve disagreements through discussions throughout the selection process. A third reviewer will make the final decision if the two paired reviewers cannot resolve the disagreement.

The search results and the study selection process will be reported in the final scoping review and presented in a PRISMA extension for scoping review (PRISMA-ScR) flow diagram.(25) All data will be recorded and exported into Excel form after the whole process ends.

## Data extraction

Data will be extracted from literature included in the scoping review by two reviewers independently using a tailored data extraction tool developed by the authors. If discrepancies occur during the data extraction process, the two reviewers will discuss to reach a common decision. If there is an unsolved disagreement, a third reviewer will make the final decision. A pilot test will be conducted to ensure consistency among the reviewers. **Table 3** presents the draft of the data extraction form.

The authors might modify the draft data extraction form during data extraction. The scoping review will detail the modifications compared with this protocol.

For peer review only

**Table 3**. Data extraction

| **Extraction category** | **Description** |
|---|---|
| Name | Full name of the index or assessment tool |
| Developer | Author or agency that developed the index or assessment tool |
| Source of information | Websites or literature as the information portal for the index or assessment tool |
| Operation, if applicable | Roles and coordination among sponsor, funder, manager or other stakeholders |
| Domain | Domain in health, such as health system, health security, health information, healthcare quality |
| Objectives | The purpose for index or assessment tool creation; the assessed subjects. |
| Geographic coverage | Number and the geographic regions (e.g., Asia) of countries assessed |
| Time coverage | Year(s) of publication or the time frame the index or assessment tool being used |
| Implementation level | The implementation level that the index or assessment tool was designed to assess (i.e., global, national, subnational or local level) |
| Dimensions | The dimensions (not the specific indicators) of assessment content |
| Theory or logic, if applicable | The theory or logic based to develop the index or assessment tool |
| Data sources | The approach used to obtain information necessary for the assessment, such as self-assessment and open-source data. |
| Methods | Qualitative, quantitative or mixed methods |
| Types of assessment results (if there are any open ones) | Scores or other types of results that present the assessment results |
| Validity and reliability, if applicable | Description of the validation process or reliability check of the assessment |

## Data analysis and presentation

Data analysis will be qualitative, following the data extraction form to explicate further and compare each index or assessment tool. NVivo will be used in the coding process to analyse the specific indicators, and the role of "governance" will be particularly specified. This scoping review will also dialogue with existing critical analysis on the index and assessment tools eligible for this study. As a practical contribution of this scoping review, a feasibility and necessity assessment will be conducted in the discussion section, identifying the strengths and limitations of existing indexes and assessment tools to inform the future research and application of the global health governance index or consensus framework development.

## Patient and public involvement

Patients and the public will not be involved in this scoping review.



For peer review only - http://bmjopen.bmj.com/site/about/guidelines.xhtml

# ETHICS AND DISSEMINATION

The analytical results will inform various stakeholders, including researchers, public health agencies, governments, global health organisations, and other health governance actors. Dissemination of this scoping review will include publication in a peer-reviewed scientific journal, policy briefs and conference presentations. Ethics approval is not required as the data are available publicly.

## Acknowledgements

The authors are grateful to two librarians, Muriel Leclerc from the University of Geneva and Yi Ren from Tsinghua University, for their guidance on the search strategy. Also, thank Marhaba Abdurëhim, Junpeng Wang, and Yunke Gu for doing the initial search to check the availability of the potential indexes or assessment tools.

## Author Contributions

All authors contributed to the study's design, drafted the manuscript, provided feedback, and approved the final manuscript. KT provided feedback in principle, oversaw revisions and refined the manuscript. KT will also be the guarantor of the review. AH developed the search strategy, eligibility criteria, and data extraction tool and drafted and edited the protocol. YL structured the protocol, drafted the methods, refined the search strategy and eligibility criteria in detail, and contributed to the pilot tests. LZ drafted the introduction session and the supplemental material, refined the search strategy and the manuscript, and contributed to the pilot tests. JD developed the initial search strategy and eligibility criteria and drafted the methods. QH led the initial search to check the availability of the potential indexes or assessment tools.

## Funding

This work is supported by the Research Fund of Vanke School of Public Health, Tsinghua University (No. 2021ZZ004) and Spring Breeze Fund, Tsinghua University (No. 20203080035). The funders are not involved in the research. They will also have no input on the interpretation or publication of the study results.

## Competing interests

None declared.

## Patient consent for publication

None declared.

## Data statement

The data is not applicable as this article is only a protocol. For the scoping review, the data will be accessed in an open data repository. When requested, the authors will provide missing or additional data.

For peer review only

# REFERENCES

1 Chhotray V, Stoker G. Governance: from theory to practice. In: Chhotray V, Stoker G, eds. *Governance Theory and Practice: A Cross-Disciplinary Approach*. London: Palgrave Macmillan UK 2009. 214–47. doi:10.1057/9780230583344_10

2 Rhodes RAW. Understanding governance: ten years on. *Organ Stud* 2007;**28**:1243–64. doi:10.1177/0170840607076586

3. World Health Organization. Monitoring the building blocks of health systems: a handbook of indicators and their measurement strategies [Internet]. Geneva: World Health Organization; 2010 [cited 2022 Mar 23]. Available from: https://apps.who.int/iris/handle/10665/258734

4 Frenk J, Moon S. Governance challenges in global health. *N Engl J Med* 2013;**368**:936–42. doi:10.1056/NEJMra1109339

5 de Campos TC. Guiding principles of global health governance in times of pandemics: solidarity, subsidiarity, and stewardship in COVID-19. *Am J Bioeth* 2020;**20**:212–4. doi:10.1080/15265161.2020.1779862

6 Gostin LO, Moon S, Meier BM. Reimagining global health governance in the age of COVID-19. *Am J Public Health* 2020;**110**:1615–9. doi:10.2105/AJPH.2020.305933

7 West-Oram PGN, Buyx A. Global health solidarity. *Public Health Ethics* 2017;**10**:212–24. doi:10.1093/phe/phw021

8. Global Health 50/50. The global health 50/50 report 2020: power, privilege and priorities. London, UK; 2020.

9. Larson C. Global health indicators: an overview. Can Med Assoc J. 2004 Nov 9;171(10):1199–200.

10. Kirigia JM, Kirigia DG. The essence of governance in health development. Int Arch Med. 2011 Mar 28;4(1):11.

11. Peters MDJ, Marnie C, Tricco AC, Pollock D, Munn Z, Alexander L, et al. Updated methodological guidance for the conduct of scoping reviews. JBI Evid Implement. 2021 Mar;19(1):3–10.

12. Munn Z, Peters MDJ, Stern C, Tufanaru C, McArthur A, Aromataris E. Systematic review or scoping review? Guidance for authors when choosing between a systematic or scoping review approach. BMC Med Res Methodol. 2018 Nov 19;18(1):143.

13. Mohammed K, Ahmed B. The governance of basic health coverage: A systematic review. Ethiop J Health Dev [Internet]. 2020 Oct 22 [cited 2021 Nov 20];34(3). Available from: https://www.ajol.info/index.php/ejhd/article/view/201342

14 Ntr A, M RA, C LP. The Literature review of the governance frameworks in health system. *J Public Adm Gov* 2019;**9**:252. doi:10.5296/jpag.v9i3.15535

15. Pyone T, Smith H, van den Broek N. Frameworks to assess health systems governance: a systematic review. Health Policy Plan. 2017 Jun 1;32(5):710–22.

For peer review only

16. Ravi SJ, Meyer D, Cameron E, Nalabandian M, Pervaiz B, Nuzzo JB. Establishing a theoretical foundation for measuring global health security: a scoping review. BMC Public Health. 2019 Jul 17;19(1):954.

17. Assefa Y, Nigusie A, Gedle D, Debie A. An integrated health system resilience framework to realize universal health coverage and health security: a systematic review. PROSPERO; 2020. Report No.: CRD42020210471.

18. Debie A, Khatri R, Assefa Y. Successes and challenges of health system governance towards universal health coverage and global health security: a systematic review [Internet]. PROSPERO; 2021 [cited 2021 Nov 18]. Report No.: CRD42021270090. Available from: https://www.crd.york.ac.uk/prospero/display_record.php?RecordID=270090

19. Mikkelsen-Lopez I, Wyss K, de Savigny D. An approach to addressing governance from a health system framework perspective. BMC Int Health Hum Rights. 2011 Dec 2;11(1):13.

20. Fehr R, Alexanderson K, Favaretti C, de Jong J, La Torre G, Lim T-A, et al. Health assessments for health governance—concepts and methodologies. Eur J Public Health. 2017 Aug 1;27(4):609–16.

21 Haeberer M, Tsolova S, Riley P, *et al.* Tools for assessment of country preparedness for public health emergencies: a critical review. *Disaster Med Public Health Prep* 2021;**15**:431–41. doi:10.1017/dmp.2020.13

22. Chiossi S, Tsolova S, Ciotti M. Assessing public health emergency preparedness: A scoping review on recent tools and methods. Int J Disaster Risk Reduct. 2021 Apr 1;56:102104.

23. Arksey H, O'Malley L. Scoping studies: towards a methodological framework. Int J Soc Res Methodol. 2005 Feb;8(1):19–32.

24 Peters M, Godfrey C, McInerney P, *et al.* Scoping reviews. In: Aromataris E, Munn Z, eds. *JBI Manual for Evidence Synthesis*. JBI 2020. doi:10.46658/JBIMES-20-12

25 Tricco AC, Lillie E, Zarin W, *et al.* PRISMA extension for scoping reviews (PRISMA-ScR): checklist and explanation. *Ann Intern Med* 2018;**169**:467–73. doi:10.7326/M18-0850

26. Page MJ, Moher D, Bossuyt PM, Boutron I, Hoffmann TC, Mulrow CD, et al. PRISMA 2020 explanation and elaboration: updated guidance and exemplars for reporting systematic reviews. BMJ. 2021 Mar 29;n160.

27. Rethlefsen ML, Kirtley S, Waffenschmidt S, Ayala AP, Moher D, Page MJ, et al. PRISMA-S: an extension to the PRISMA Statement for Reporting Literature Searches in Systematic Reviews. Syst Rev. 2021 Jan 26;10(1):39.

28. Shamseer L, Moher D, Clarke M, Ghersi D, Liberati A, Petticrew M, et al. Preferred reporting items for systematic review and meta-analysis protocols (PRISMA-P) 2015: elaboration and explanation. Res METHODS. 2015;25.

29. Moher D, Shamseer L, Clarke M, Ghersi D, Liberati A, Petticrew M, et al. Preferred reporting items for systematic review and meta-analysis protocols (PRISMA-P) 2015 statement. Syst Rev. 2015 Dec;4(1):1.

For peer review only

30. McGowan J, Sampson M, Salzwedel DM, *et al.* PRESS Peer Review of Electronic Search Strategies: 2015 guideline statement. *J Clin Epidemiol* 2016;**75**:40–6. doi:10.1016/j.jclinepi.2016.01.021

For peer review only

# Appendix A to Appendix D

## Appendix A. KEY CONCEPTS

### Governance

Governance has been studied in various dimensions, such as socio-legal studies, political science, economics, and development studies. In a broader term, governance is described as a series of collective actions and decision-making procedures with diverse actors and organisations without formal control mechanisms. Governance does not depend on authority and coercion; it is achieved through negotiation, communication, and hegemonic influence.(1) It does not only concern the government, and it emphasises governing with and through networks between public, private and voluntary sectors.(2) The United Nations Development Programme (UNDP) refers to good governance broadly as the principles of legitimacy and voice, direction, performance, accountability, and fairness.(3) Definition of governance World Health Organization (WHO) focuses on effective oversight, coalition-building, the provision of regulations and incentives, attention to system-design and accountability.(4) Concerning health, Baez-Camargo and Jacobs define governance as "processes through which health systems manage human resources, acquire and distribute medicines and technologies, generate and disseminate information, and provide means to finance the provision of health services to the population."(5) Given the inclusiveness of "governance", this scoping review does not aim to define a concrete *priori* concept of governance. Rather, it tried extensively potential search terms according to the existing analysis of governance dimensions (**Table A**).[1]

Based on Table A, we develop the search terms for the concept "governance" in our search strategy. The search terms cover all the dimensions or search terms for "governance" overlapped in the included review listed in Table A. Terms with similar meaning are classified in one group, such as participation and involvement. However, the silo terms not overlapping are not included as search terms in this review.

Besides, we also referred to search strategies of existing systematic reviews or scoping reviews on health governance.(9–15) Apart from "governance" itself and the dimensions listed above, we add another term, "leadership", which has been widely used as a search term for the concept of "governance".

[1] We used the search strings “governance AND (concepts OR concept OR definition* OR define OR defining OR meaning*)” in Web of Science, Ovid (Embase <1974 to 2022 March 11> and Medline and Epub Ahead of Print, In-Process, In-Data-Review & Other Non-Indexed Citations, Daily and Versions <1946 to March 11, 2022>), Cochrane, and Google Scholar and looked for reviews (systematic/scoping or systematic-like reviews) with summarized dimensions of “governance”. The search field was “Title” in bibliographic databases, and the time frame was 2000-2021. In order to find additional literature to the bibliographic databases, there is no search field in Google Scholar and the top 100 items by relevance were screened. The search date was 14 March 2022. In Barbazza et al. (2014), broad dimensions include some fundamental values and outcomes that could be independent from the concept of governance, so we only list the functional dimensions according to this article in Table A.

For peer review only - http://bmjopen.bmj.com/site/about/guidelines.xhtml

**Table A**. Summary of review regarding governance dimensions

| *Classified dimensions or search terms for "governance"* | *Carlson et al., "Defining the functions of public health governance." American Journal of Public Health (2015).(6)* | *Barbazza and Tello, "A review of health governance: definitions, dimensions and tools to govern." Health Policy (2014). (7)* | *Ruhanen et al. "Governance: a review and synthesis of the literature." Tourism Review (2010).(8)* |
|---|---|---|---|
| *Accountability* | | Accountability | Accountability |
| *Stewardship* | Resource Stewardship | Stewardship | |
| *Transparency* | | Transparency | Transparency |
| *Policy* | Policy development | Formulating policy/strategic direction | |
| *strategic* | | Formulating policy/strategic direction | Strategic vision |
| *Partner/partnership* | Partner Engagement | Partnerships | |
| *Participation/involvement* | | Participation and consensus | Involvement |
| *Consensus* | | Participation and consensus | Consensus |

## Global Health Governance

While titled "health governance across countries", this scoping review is embedded in the context of global health governance. Although existing literature has centred on governance in health systems and health development only in recent decades, there has been an increasing interest in discussing the relationship between governance and global health. Despite the extensive scholarly debates on the definition of global health, it is still elusive to reach a consensus around a precise definition. In 2009, Koplan and colleagues argued for "a common definition of global health", which emphasises transnational health, and embraces different disciplines and interdisciplinary collaboration.(16) Scholars delineate global health by focusing on multiple dimensions, such as education, governance, security, etc. Many also view global health as a mode of governance across borders.(17) The term global health governance (GHG) is widely used in scholarly work, but few researchers agree on how the term should be applied. Lee and Kamradt-Scott point out that the GHG peer-reviewed literature varies substantially on what kinds of and to what extent institutions should engage in GHG. In addition, the goal and function of GHG are also not clear.(18) For instance, Kickbusch and Szabo refer GHG mainly to institutions and governance processes that are directly linked to health, such as the WHO.(19) Another highly cited definition proposed by David P. Fidler takes a more inclusive approach. He defines GHG as "the use of formal and informal institutions, rules, and processes by states, intergovernmental organisations, and non-state actors to deal with challenges to health that require cross-border collective action to address effectively." (20)

# Appendix B. PRELIMINARY SEARCHES

The methods for searching related published/ongoing reviews were: (1) directly searching using strings "'health governance' AND ('index' OR 'indicator' OR assess) AND ('systematic


For peer review only - http://bmjopen.bmj.com/site/about/guidelines.xhtml

review' OR 'scoping review')"; (2) forward snowballing for the reviews including our potential eligible literature such as GHSI and IHR core capacity, using strings "('global health security index' OR 'GHSI') AND ('systematic review' OR 'scoping review')" and "('international health regulations' OR 'IHR') AND ('systematic review' OR 'scoping review') AND ('core capacity' OR 'score')". There was no filter or limit and the search dates were 20 November 2021 and 22 March 2022.

# Appendix C. FULL SEARCH STRATEGY

## Web of science Core Collection

Search date: 03 April 2022

| *No.* | *Query* | *Results* |
|---|---|---|
| #1 | TS=(“health” OR “healths” OR "healthcare*") | 2,899,246 |
| #2 | TS=("governance*" OR "leadership*" OR "accountabilit*" OR "stewardship*" OR (("policy" OR "policies") NEAR/10 (“develop” OR “developing” OR “develops” OR “developed” OR “development” OR “developments” OR “formulat*”)) OR ("strateg*" NEAR/10 (“vision” OR “visions” OR “direction” OR “directions”)) OR "partner*" OR "transparen*" OR "participation*" OR "involvement*" OR "consensus*") | 2,178,485 |
| #3 | TS=("evaluate" OR "evaluated" OR "evaluates" OR "evaluating" OR "evaluation*" OR "monitor" OR "monitors" OR "monitored" OR "monitoring" OR "measure " OR "measures" OR "measured" OR "measuring" OR "measurement*" OR "assess" OR "assesses" OR "assessed" OR "assessing" OR "assessment*") | 15,885,119 |
| #4 | TS=("indicator*" OR "score" OR "scores" OR "scored" OR "scoring" OR "index" OR "indexs" OR "indexes" OR "indexed" OR "indices" OR "indexing") | 3,259,711 |
| #5 | TS=("globe" OR "global" OR "globally" OR "international" OR "internationally" OR "world" OR "worldwide" OR "worldwidely" OR "multi country " OR "multi countries”) | 3,745,205 |
| #6 | #1 AND #2 AND #3 AND #4 AND #5 AND DOP=(2000-01-01/2021-12-31) | 8,046 |
| #7 | #7 NOT DT=(Review OR Editorial Material OR Meeting Abstract OR Letter) | 6,844 |

## PubMed

Search date: 03 April 2022

| *No* | *Query* | *Results* |
|---|---|---|
| #1 | "health"[MeSH Terms] OR "health"[Title/Abstract] OR "healths"[Title/Abstract] OR "healthcare*"[Title/Abstract] | 2,617,637 |
| #2 | "leadership"[MeSH Terms] OR "public policy"[MeSH Terms] OR "consensus"[MeSH Terms] OR "social participation"[MeSH Terms] | 1,434,193 |

| | | |
|---|---|---|
| | OR "community participation"[MeSH Terms] OR "stakeholder participation"[MeSH Terms] OR "governance*"[Title/Abstract] OR "leadership*"[Title/Abstract] OR "accountabilit*"[Title/Abstract] OR "stewardship*"[Title/Abstract] OR (("policy"[Title/Abstract] OR "policies"[Title/Abstract]) AND (("develop"[Title/Abstract] OR "developing"[Title/Abstract] OR "develops"[Title/Abstract] OR "developed"[Title/Abstract] OR "development"[Title/Abstract] OR "developments"[Title/Abstract]) OR "formulat*"[Title/Abstract])) OR ("strateg*"[Title/Abstract] AND ("vision"[Title/Abstract] OR "visions"[Title/Abstract] OR "direction"[Title/Abstract] OR "directions"[Title/Abstract])) OR "partner*"[Title/Abstract] OR "transparen*"[Title/Abstract] OR "participation*"[Title/Abstract] OR "involvement*"[Title/Abstract] OR "consensus*"[Title/Abstract] | |
| #3 | "evaluate"[Title/Abstract] OR "evaluated"[Title/Abstract] OR "evaluates"[Title/Abstract] OR "evaluating"[Title/Abstract] OR "evaluation*"[Title/Abstract] OR "monitor"[Title/Abstract] OR "monitors"[Title/Abstract] OR "monitored"[Title/Abstract] OR "monitoring"[Title/Abstract] OR "measure"[Title/Abstract] OR "measures"[Title/Abstract] OR "measured"[Title/Abstract] OR "measuring"[Title/Abstract] OR "measurement*"[Title/Abstract] OR "assess"[Title/Abstract] OR "assesses"[Title/Abstract] OR "assessed"[Title/Abstract] OR "assessing"[Title/Abstract] OR "assessment*"[Title/Abstract] | 9,147,656 |
| #4 | "indicator*"[Title/Abstract] OR "score"[Title/Abstract] OR "scores"[Title/Abstract] OR "scored"[Title/Abstract] OR "scoring"[Title/Abstract] OR "index"[Title/Abstract] OR "indexs"[Title/Abstract] OR "indexes"[Title/Abstract] OR "indexed"[Title/Abstract] OR "indices"[Title/Abstract] OR "indexing"[Title/Abstract] | 2,311,393 |
| #5 | "globe"[Title/Abstract] OR "global"[Title/Abstract] OR "globally"[Title/Abstract] OR "international"[Title/Abstract] OR "internationally"[Title/Abstract] OR "world"[Title/Abstract] OR "worldwide"[Title/Abstract] OR "worldwidely"[Title/Abstract] OR "multi country"[Title/Abstract] OR "multi countries"[Title/Abstract] | 1,490,795 |
| #6 | #1 AND #2 AND #3 AND #4 AND #5 | 7,928 |
| #7 | #1 AND #2 AND #3 AND #4 AND #5 AND ("2000/01/01"[Date - Publication] : "2021/12/31"[Date - Publication]) | 7,407 |
| #8 | (#7) NOT (Comment[Publication Type] OR Editorial[Publication Type] OR Letter[Publication Type] OR Review[Publication Type]) | 6,270 |

## Embase

Search date: 03 April 2022

| *No.* | *Query* | *Results* |
|---|---|---|
| #1 | 'health'/exp OR 'public health'/exp OR 'health care'/exp OR health:ab,ti,kw OR healths:ab,ti,kw OR 'healthcare*':ab,ti,kw | 11,573,270 |
| #2 | 'leadership'/exp OR 'policy'/exp OR 'consensus'/exp OR 'governance'/exp OR 'accountability'/exp OR 'strategy'/exp OR 'partner'/exp OR 'partnership'/exp OR 'participation'/exp OR | 1,866,006 |



For peer review only - http://bmjopen.bmj.com/site/about/guidelines.xhtml

| | | |
|---|---|---|
| | 'involvement'/exp OR 'transparency'/exp OR 'governance*':ab,ti,kw OR 'leadership*':ab,ti,kw OR 'accountabilit*':ab,ti,kw OR 'stewardship*':ab,ti,kw OR (('policy' OR 'policies') NEAR/10 ('develop' OR 'developing' OR 'develops' OR 'developed' OR 'development' OR 'developments' OR 'formulat*')):ab,ti,kw OR ('strateg*' NEAR/10 ('vision' OR 'visions' OR 'direction' OR 'directions')):ab,ti,kw OR 'partner*':ab,ti,kw OR 'transparen*':ab,ti,kw OR 'participation*':ab,ti,kw OR 'involvement*':ab,ti,kw OR 'consensus*':ab,ti,kw | |
| #3 | 'monitor'/de OR 'measurement'/de OR 'assessment'/exp OR evaluate:ab,ti,kw OR evaluated:ab,ti,kw OR evaluates:ab,ti,kw OR evaluating:ab,ti,kw OR evaluation*:ab,ti,kw OR monitor:ab,ti,kw OR monitors:ab,ti,kw OR monitored:ab,ti,kw OR monitoring:ab,ti,kw OR measure:ab,ti,kw OR measures:ab,ti,kw OR measured:ab,ti,kw OR measuring:ab,ti,kw OR measurement*:ab,ti,kw OR assess:ab,ti,kw OR assesses:ab,ti,kw OR assessed:ab,ti,kw OR assessing:ab,ti,kw OR assessment*:ab,ti,kw | 12,334,482 |
| #4 | 'indicator'/de OR 'score'/exp OR 'index'/exp OR indicator*:ab,ti,kw OR score:ab,ti,kw OR scores:ab,ti,kw OR scored:ab,ti,kw OR scoring:ab,ti,kw OR index:ab,ti,kw OR indexs:ab,ti,kw OR indexes:ab,ti,kw OR indexed:ab,ti,kw OR indices:ab,ti,kw OR indexing:ab,ti,kw | 3,358,824 |
| #5 | 'global'/exp OR 'world'/exp OR globe:ab,ti,kw OR global:ab,ti,kw OR globally:ab,ti,kw OR international:ab,ti,kw OR internationally:ab,ti,kw OR world:ab,ti,kw OR worldwide:ab,ti,kw OR worldwidely:ab,ti,kw OR 'multi country':ab,ti,kw OR 'multi countries':ab,ti,kw | 2,099,845 |
| #6 | #1 AND #2 AND #3 AND #4 AND #5 | 19,014 |
| #7 | #1 AND #2 AND #3 AND #4 AND #5 AND [2000-2021]/py | 18,199 |
| #8 | #7 NOT (editorial:it OR letter:it OR review:it OR 'conference abstract':it) | 9,416 |

## Google

Search date: 03 April 2022

Customed range: 2000-2021

| *No.* | *Query (Google limits queries to 32 words)* | *Results* |
|---|---|---|
| *1* | health AND (governance OR leadership OR accountability OR stewardship) AND (evaluate OR monitor OR measure OR assess) AND (index OR indicator OR score) AND (global OR international OR world OR multi-country) | 151 |
| *2* | health AND (transparency OR policy OR strategy) AND (evaluate OR monitor OR measure OR assess) AND (index OR indicator OR score) AND (global OR international OR world OR multi-country) | 178 |



For peer review only - http://bmjopen.bmj.com/site/about/guidelines.xhtml

| | | |
|---|---|---|
| *3* | health AND (partnership OR participation OR involvement OR consensus) AND (evaluate OR monitor OR measure OR assess) AND (index OR indicator OR score) AND (global OR international OR world OR multi-country) | 157 |

## Appendix D. ELIGIBILITY CRITERIA IN DETAIL

| | *Inclusion criteria* | *Exclusion criteria* |
|---|---|---|
| *Subjects* | Indexes or assessment tools for health governance with measurable indicators assessing health governance, including but not limited to:<br><br>1.1 General health governance;<br>1.2 Governance of health system;<br>1.3 Governance of public health risks such as public health emergencies, epidemics and pandemics;<br>1.4 Governance on specific health issues such as influenza, reproductive health, etc. | Assessment/conceptual frameworks, or narrative assessment without measurement; on topics irrelevant to health<br>1.1 Not being an index or assessment tool;<br>1.2 Irrelevant to our topics<br>1.2.1 Not governance-related;<br>1.2.2 Not health-related;<br>1.2.3 Health outcome-based;<br>1.2.4 Both non-governance- and non-health-related |
| *Objectives* | 2 Describing the indexes or assessment tools (incl. indicators or scoring results) | 2 Only criticising, mentioning, analysing the indexes or assessment tools while not aiming to yield assessment results for health governance |
| *Coverage* | 3 Can be applied in multi-countries at global level (although the assessed subject might be sub-national/national entities) | 3.1 Geographical coverage: Only applied/can be applied within one country or one region;<br>3.2 Entity coverage: Assessing one type of specific organisations or individuals (e.g., hospital or enterprise)<br>3.3 Case study: Appearing as a case study without further generalisation |
| *Type of sources* | 4 Reports, documents, peer-reviewed publications, websites | 4 Wrong publication type: commentaries, reviews, blogs, protocols and so on |



For peer review only - http://bmjopen.bmj.com/site/about/guidelines.xhtml

# REFERENCES

1. Chhotray V, Stoker G. Governance: From Theory to Practice. In: Chhotray V, Stoker G, editors. Governance Theory and Practice: A Cross-Disciplinary Approach [Internet]. London: Palgrave Macmillan UK; 2009 [cited 2022 Mar 23]. p. 214–47. Available from: https://doi.org/10.1057/9780230583344_10

2. Rhodes RAW. Understanding Governance: Ten Years On. Organ Stud. 2007 Aug;28(8):1243–64.

3. Nations DPU. Governance for sustainable human development: A UNDP policy document. UNDP; 1997.

4. Organisation WH. Everybody's business–strengthening health systems to improve health outcomes: WHO's framework for action. 2007;

5. Baez Camargo C, Jacobs E. A framework to assess governance of health systems in low income countries. 2011;

6. Carlson V, Chilton MJ, Corso LC, Beitsch LM. Defining the Functions of Public Health Governance. Am J Public Health. 2015 Apr;105(S2):S159–66.

7. Barbazza E, Tello JE. A review of health governance: Definitions, dimensions and tools to govern. Health Policy. 2014 May 1;116(1):1–11.

8. Ruhanen L, Scott N, Ritchie B, Tkaczynski A. Governance: a review and synthesis of the literature. Pechlaner H, Raich F, Beritelli P, editors. Tour Rev. 2010 Jan 1;65(4):4–16.

9. Hastings S, Armitage G, Mallinson S, Jackson K, Suter E. Exploring the relationship between governance mechanisms in healthcare and health workforce outcomes: a systematic review. BMC Health Serv Res. 2014 Oct 4;14.

10. Pyone T, Smith H, van den Broek N. Frameworks to assess health systems governance: a systematic review. Health Policy Plan. 2017 Jun 1;32(5):710–22.

11. Abd Rahim A, Manaf R, Juni M, Ibrahim N. Health System Governance for the Integration of Mental Health Services into Primary Health Care in the Sub-Saharan Africa and South Asia Region: A Systematic Review. Inq- J Health CARE Organ Provis Financ. 2021 Jul;58.

12. Mohammed K, Ahmed B. The governance of basic health coverage: A systematic review. Ethiop J Health Dev [Internet]. 2020 Oct 22 [cited 2021 Nov 20];34(3). Available from: https://www.ajol.info/index.php/ejhd/article/view/201342

13. Naher N, Hoque R, Hassan M, Balabanova D, Adams A, Ahmed S. The influence of corruption and governance in the delivery of frontline health care services in the public sector: a scoping review of current and future prospects in low and middle-income countries of south and south-east Asia. BMC PUBLIC Health. 2020 Jun 8;20(1).

14. Ayanore M, Amuna N, Aviisah M, Awolu A, Kipo-Sunyehzi D, Mogre V, et al. Towards Resilient Health Systems in Sub-Saharan Africa: A Systematic Review of the English Language Literature on Health Workforce, Surveillance, and Health Governance Issues for Health Systems Strengthening. Ann Glob Health. 2019;85(1).

For peer review only

15. Tabrizi J.S., Pourasghar F., Nikjoo R.G. Primary health care governance pillars and components in developed countries, a systematic review. Int J Pharm Technol. 2016;8(4):24045–59.

16. Koplan JP, Bond TC, Merson MH, Reddy KS, Rodriguez MH, Sewankambo NK, et al. Towards a common definition of global health. The Lancet. 2009 Jun 6;373(9679):1993–5.

17. Salm M, Ali M, Minihane M, Conrad P. Defining global health: findings from a systematic review and thematic analysis of the literature. BMJ Glob Health. 2021;6:1–14.

18. K L, A K-S. The multiple meanings of global health governance: A call for conceptual clarity. Glob Health. 1744th–8603rd ed. 2014 Apr;10(1):no pagination.

19. Kickbusch I, Szabo MMC. A new governance space for health. Glob Health Action. 2014 Feb 13;7(s2):23507.

20. Kickbusch I. Global Health Governance Challenges 2016 – Are We Ready? Int J Health Policy Manag. 2016 Feb 29;5(6):349–53.

For peer review only

# Reporting checklist for protocol of a systematic review and meta analysis.

Based on the PRISMA-P guidelines.

## Instructions to authors

Complete this checklist by entering the page numbers from your manuscript where readers will find each of the items listed below.

Your article may not currently address all the items on the checklist. Please modify your text to include the missing information. If you are certain that an item does not apply, please write "n/a" and provide a short explanation.

Upload your completed checklist as an extra file when you submit to a journal.

In your methods section, say that you used the PRISMA-Preporting guidelines, and cite them as:

Moher D, Shamseer L, Clarke M, Ghersi D, Liberati A, Petticrew M, Shekelle P, Stewart LA. Preferred Reporting Items for Systematic Review and Meta-Analysis Protocols (PRISMA-P) 2015 statement. Syst Rev. 2015;4(1):1.

| | | Reporting Item | Page Number |
|---|---|---|---|
| **Title** | | | |
| Identification | #1a | Identify the report as a protocol of a systematic review | 1 |
| Update | #1b | If the protocol is for an update of a previous systematic review, identify as such | N/A. Not an update. |

For peer review only

| | | | |
|---|---|---|---|
| **Registration** | | | |
| | #2 | If registered, provide the name of the registry (such as PROSPERO) and registration number | 5 |
| **Authors** | | | |
| Contact | #3a | Provide name, institutional affiliation, e-mail address of all protocol authors; provide physical mailing address of corresponding author | 1 |
| Contribution | #3b | Describe contributions of protocol authors and identify the guarantor of the review | 9 |
| **Amendments** | | | |
| | #4 | If the protocol represents an amendment of a previously completed or published protocol, identify as such and list changes; otherwise, state plan for documenting important protocol amendments | 5 |
| **Support** | | | |
| Sources | #5a | Indicate sources of financial or other support for the review | 10 |
| Sponsor | #5b | Provide name for the review funder and / or sponsor | 10 |
| Role of sponsor or funder | #5c | Describe roles of funder(s), sponsor(s), and / or institution(s), if any, in developing the protocol | 10 |
| **Introduction** | | | |

For peer review only

| | | | |
|---|---|---|---|
| Rationale | #6 | Describe the rationale for the review in the context of what is already known | 3 |
| Objectives | #7 | Provide an explicit statement of the question(s) the review will address with reference to participants, interventions, comparators, and outcomes (PICO) | 3-4 |
| **Methods** | | | |
| Eligibility criteria | #8 | Specify the study characteristics (such as PICO, study design, setting, time frame) and report characteristics (such as years considered, language, publication status) to be used as criteria for eligibility for the review | 4,7 |
| Information sources | #9 | Describe all intended information sources (such as electronic databases, contact with study authors, trial registers or other grey literature sources) with planned dates of coverage | 5-6 |
| Search strategy | #10 | Present draft of search strategy to be used for at least one electronic database, including planned limits, such that it could be repeated | 5-6 |
| Study records - data management | #11a | Describe the mechanism(s) that will be used to manage records and data throughout the review | 7 |
| Study records - selection process | #11b | State the process that will be used for selecting studies (such as two independent reviewers) through | 7 |

For peer review only

| | | | |
|---|---|---|---|
| | | each phase of the review (that is, screening, eligibility and inclusion in meta-analysis) | |
| Study records - data collection process | #11c | Describe planned method of extracting data from reports (such as piloting forms, done independently, in duplicate), any processes for obtaining and confirming data from investigators | 8 |
| Data items | #12 | List and define all variables for which data will be sought (such as PICO items, funding sources), any pre-planned data assumptions and simplifications | N/A. Not a systematic review protocol. |
| Outcomes and prioritization | #13 | List and define all outcomes for which data will be sought, including prioritization of main and additional outcomes, with rationale | N/A. Not a systematic review protocol. |
| Risk of bias in individual studies | #14 | Describe anticipated methods for assessing risk of bias of individual studies, including whether this will be done at the outcome or study level, or both; state how this information will be used in data synthesis | N/A. Not a systematic review protocol. |
| Data synthesis | #15a | Describe criteria under which study data will be quantitatively synthesised | N/A. Not a quantitative scoping review protocol. |
| Data synthesis | #15b | If data are appropriate for quantitative synthesis, describe planned summary measures, methods of handling data and methods of combining data from studies, including any planned exploration of | N/A. Not a quantitative scoping review protocol. |

For peer review only

| | | | |
|---|---|---|---|
| | | consistency (such as I2, Kendall's τ) | |
| Data synthesis | #15c | Describe any proposed additional analyses (such as sensitivity or subgroup analyses, meta-regression) | N/A. Not a quantitative scoping review protocol. |
| Data synthesis | #15d | If quantitative synthesis is not appropriate, describe the type of summary planned | 8-9 |
| Meta-bias(es) | #16 | Specify any planned assessment of meta-bias(es) (such as publication bias across studies, selective reporting within studies) | N/A. Not a systematic review protocol. |
| Confidence in cumulative evidence | #17 | Describe how the strength of the body of evidence will be assessed (such as GRADE) | N/A. Not a systematic review protocol. |

For peer review only

BMJ Open

# Assessing Health Governance Across Countries: A Scoping Review Protocol on Indices and Assessment Tools Applied Globally



| | |
|---|---|
| Journal: | *BMJ Open* |
| Manuscript ID | bmjopen-2022-063866.R1 |
| Article Type: | Protocol |
| Date Submitted by the Author: | 17-Jun-2022 |



| | |
|---|---|
| Complete List of Authors: | Huang, Aidan; Tsinghua University, Vanke School of Public Health; Tsinghua University, Institute for Healthy China<br>Lin, Yuling; University of Geneva Global Studies Institute<br>Zhang, Liyuan; University of Cambridge<br>Dong, Jingwen; Shanghai Jiao Tong University School of Public Health<br>He, Qiwei; Tsinghua University, Vanke School of Public Health; Tsinghua University, Institute for Healthy China<br>Tang, Kun; Tsinghua University, Vanke School of Public Health; Tsinghua University, Institute for Healthy China |



| | |
|---|---|
| <b>Primary Subject Heading</b>: | Health policy |
| Secondary Subject Heading: | Public health |
| Keywords: | Health policy < HEALTH SERVICES ADMINISTRATION & MANAGEMENT, PUBLIC HEALTH, Public health < INFECTIOUS DISEASES |
| | |


SCHOLARONE™
Manuscripts

# Assessing Health Governance Across Countries: A Scoping Review Protocol on Indices and Assessment Tools Applied Globally

**Aidan Huang[1], Yuling Lin[2], Liyuan Zhang[3], Jingwen Dong[4], Qiwei He[5], Kun Tang[6]**

1. Vanke School of Public Health, Tsinghua University, Beijing, China; Institute for Healthy China, Tsinghua University, Beijing, China. Email: adhuang@mail.tsinghua.edu.cn
2. Global Studies Institute, University of Geneva, 1205 Geneva, Switzerland. Email: yuling.lin@etu.unige.ch
3. University of Cambridge, Cambridge CB2 1TN, United Kingdom. Email: lz449@cam.ac.uk
4. School of Public Health, Shanghai Jiao Tong University, Shanghai, China. Email: ilovemath@sjtu.edu.cn
5. Vanke School of Public Health, Tsinghua University, Beijing, China; Institute for Healthy China, Tsinghua University, Beijing, China. Email: heqiwei@mail.tsinghua.edu.cn

6. Vanke School of Public Health, Tsinghua University, Beijing, China; Institute for Healthy China, Tsinghua University, Beijing, China. Email: tangk@mail.tsinghua.edu.cn.

**Correspondence to Dr Kun Tang**; tangk@mail.tsinghua.edu.cn. Postal address: Vanke School of Public Health, Tsinghua University, No. 30 Shuangqing Road, Beijing, 100084, China. Telephone: +86-13671129425.

Word count: 2597

For peer review only

# Abstract

**Introduction:** Most global health indices or assessment tools focus on health outcomes rather than governance, and they have been developed primarily from the perspective of high-income countries. To benchmark global health governance for equity and solidarity, it becomes necessary to reflect on the current state of indices or assessment tools evaluating health governance across countries. This scoping review aims to (1) review the existing multi-country indices and assessment tools applied globally with measurable indicators assessing health governance; (2) summarise their differences and commonalities; (3) identify the lessons learned through analysis of their advantages and gaps; (4) evaluate the feasibility and necessity to establish a new index or consensus framework for assessing global health governance.

**Methods and analysis:** This scoping review protocol follows Arksey and O'Malley's methodological framework, the Joanna Briggs Institute (JBI) guidelines and the Preferred Reporting Items for Systematic Reviews and Meta-analyses (PRISMA) methodology for scoping reviews. Key information sources will be bibliographic databases (PubMed, Embase, and Web of Science Core Collection), grey literature and citation tracking. The time frame will be 1 January 2000 to 31 December 2021. Only indices or assessment tools that are globally applicable and provide measurable indicators of health governance will be eligible. A qualitative content analysis will follow the proposed data extraction form to explicate and compare each eligible index or assessment tool. An analysis based on a proposed preliminary evaluation framework will identify the advantages and gaps and summarise the lessons learned. This scoping review will also discuss the feasibility and necessity of developing a new global health governance index or consensus framework to inform future research and practices.

**Ethics and dissemination:** This scoping review does not require ethics approval. Dissemination will include a peer-review article, policy briefs and conference presentations. This protocol has been registered in the Open Science Framework (osf.io/y93mj).

**Keywords:** health governance; index; assessment tool; global health; scoping review protocol

# Article Summary

- This scoping review will be a prior assessment in establishing a new index or consensus framework for assessing global health governance for the post-COVID-19 era.
- This scoping review will differ from the existing reviews by incorporating governance for a wide range of health objectives and broadening geographic coverage with a global lens.
- The literature to be reviewed will include research articles and indices or assessment tools used by organisations, with theoretical and practical implications for assessing health governance.
- Pilot tests in searching and study selection and consultation with multiple librarians were conducted for the protocol development.
- With the topic being broad and interdisciplinary, the precision of the search strategy might be constrained.

For peer review only

# INTRODUCTION

## Rationale

The health governance of countries shapes global health governance. In a broad sense, governance is described as a series of collective actions and decision-making procedures with diverse actors and organisations without formal control mechanisms.(1) Governance emphasises governing with and through networks between public, private and voluntary sectors.(2) It is one of the blocks in the widely-used health systems framework formulated by the World Health Organization (WHO).(3) Given the globalised health issues, health governance in each sovereignty has been closely linked. From the pandemic of SARS to COVID-19, repeating global health crises have alerted the need for global health solidarity efforts.(4) However, there is still a lack of a solid governance framework under "international anarchy"(5–7), although United Nations' 2030 Sustainable Development Goals (SDGs) have set up goals to promote global health outcomes.

Indeed, existing indices or assessment tools in global health tend to focus on health outcomes instead of the governance elements attributed to these outcomes (see Appendix A in the supplemental material). Even within health governance, multiple parallel overlapping frameworks, assessment tools and indices for theoretical or practical purposes have created complexities. Besides, 85 per cent of global health organisations have their headquarters in Europe or North America; more than 80 per cent of the global health leaders come from high-income countries. (8) Therefore, most global health indices or assessment tools and indicators have been produced from high-income countries' perspectives, failing to reflect the other populations. Due to economic constraints and low logistic capacity, health statistics in developing countries are with varying standards and difficult-to-assess accuracy.(9) Thus, global health indicators' validity, utility, and representativeness in developing countries are questionable.(10)

The underlying standpoint of this scoping review is that with the deeply-rooted notions of sovereignty, global health governance has to be anchored around the health governance of countries. A starting point might be a consensus framework or a new, integrated index on health governance across countries globally. Thus, scoping the existing indices and assessment tools will lay a practical basis for developing an index or consensus framework to benchmark global health governance for equity and solidarity.

## Objectives

This scoping review aims to (1) review the existing multi-country indices and assessment tools applied globally with measurable indicators assessing health governance; (2) summarise their differences and commons; (3) identify the lessons learned through analysis of their advantages and gaps; and (4) assess the feasibility and necessity to establish a new index or consensus framework for assessing global health governance.

As global health governance is an emerging, multidisciplinary field, a scoping review is a more appropriate tool to "assess and understand the extent of the knowledge and identify, map, report, or discuss the characteristics or concepts".(11) By contrast, systematic reviews aiming to "answer a clinically meaningful question or provide evidence to inform practice" (12), or literature reviews with less systematic, transparent and reproducible methods will not meet the objectives above.

## Eligible literature

Only indices or assessment tools that are globally applicable and provide measurable indicators of health governance will be eligible. Indices and assessment tools are both tools for evaluation with measurable indicators. In practice, "index" is often an external evaluation tool resulting in scores or rankings, while "assessment tool" often refers to guidance or checklist for benchmarked standards (it might be called "self-assessment tool" in some cases). Regarding "health", as the One Health approach has attracted increasing attention but faced challenges in operationalisation within global health governance(13), this scoping review will include indices or assessment tools related to human, animal and environmental health.


For peer review only - http://bmjopen.bmj.com/site/about/guidelines.xhtml

International institutions, universities and think tanks might have established the majority of the potentially eligible literature, such as the Global Health Security Index (GHSI), International Health Regulations (IHR) Monitoring & Evaluation Framework, and the Ocean Health Index. Other potentially eligible literature can also be found in bibliographic databases, such as the "health development governance index".(10) In the health sector, the authors could only find indices or assessment tools to evaluate national or sub-national governance, although the assessment results might be comparable across countries under international coordination. Therefore, the authors posit that the assessment of transnational, multinational, international or global health governance might be rare. However, the authors will include the latter pieces of literature if there are any.

This scoping review excludes assessment frameworks without measurable indicators for the following reasons. First, there have been scoping reviews, systematic reviews or review protocols covering health governance frameworks in the health system(14–16), health emergencies or health security(17) or both(18,19), while few of them pragmatically concentrate on indices or assessment tools. Second, most health governance frameworks have not been applied in practice, and there is a lack of real-world evidence to validate the efficacy of these frameworks. Pyone, Smith and van den Broek found that within 16 frameworks for assessing governance in the health system, only five were applied in empirical research.(16) Mikkelsen-Lopez and her colleagues also point out that the lack of empirical work might result from unrealistic indicators and overly complicated framework design.(20)

This scoping review excludes indices or assessment tools designed to be applied in a particular country or region. Some reviews have included indices or assessment tools applied in regions like Europe as part of eligible literature(21–23). Moreover, considering the objectives of this scoping review, including indices or assessment tools applied in particular countries or regions will weaken the global generalisability. Moreover, since the concept of "governance" in this scoping review involves diverse actors and organisations, governance of only one type of organisation (e.g., hospital or enterprise) does not fit this research's scope.

## Related published/ongoing reviews

The authors did not identify any published or ongoing systematic reviews or scoping reviews on the topic through a preliminary search in Google Scholar, PROSPERO, JBI Evidence Synthesis, Figshare, Open Science Framework, and Research Gate (see Appendix B in the supplemental material for the methods of the preliminary search). Some eligible indices or assessment tools included in similar reviews(23) will be included and analysed in this scoping review, although their objectives and analytical methods differ from those of this scoping review.

Specifically, this scoping review will differ from the existing reviews by (1) incorporating governance for a wide range of health objectives, such as health system strengthening (including universal health coverage) and health security (including public health emergency preparedness); (2) broadening the geographic coverage with a global lens; (3) focusing on indices or assessment tools in practice to inform decision-making for future assessment of global health governance.

# METHODS

This scoping review protocol follows Arksey and O'Malley's methodological framework(24), the Joanna Briggs Institute (JBI) guidelines(25) and the Preferred Reporting Items for Systematic Reviews and Meta-analyses (PRISMA) methodology for scoping reviews(11,26). The reviewers also refer to systematic review methods (e.g., search strategy and reporting) that might assist the transparency and rigorousness of this scoping review.(27–31)

This protocol has been registered in the Open Science Framework (osf.io/y93mj). The searches were conducted in each proposed information source on 3 April 2022. The following research and writing will start in June 2022 and last 2-3 months. The final scoping review will report important protocol amendments and their rationales.


For peer review only - http://bmjopen.bmj.com/site/about/guidelines.xhtml

## Research questions

Following the objectives of this scoping review, the primary research question will guide the study: what indices or assessment tools are designed to assess health governance across multiple countries? Besides, two additional research questions are based on the primary question. First, what are their differences and commonalities? Second, what are the lessons learned to inform the future global health governance index or consensus framework development?

## Identifying relevant studies

### Electronic Searches

The search strategy will locate both publications in bibliographic databases and grey literature and adapt for each included information source. Given that only the term "health governance" started to appear in the published literature around 2000, the search will be filtered by the publication dates between 1 January 2000 and 31 December 2021. The Peer Review of Electronic Search Strategies (PRESS) checklist has been used for the proposed full search strategy.(31)

Our search terms come from the following sources: (1) concepts related to research questions; (2) MeSH and Emtree databases; (3) completed and ongoing related systematic reviews and scoping reviews. Using **Table 1**, the authors join all terms within each concept with OR and join each concept together using AND.

**Table 1.** Search terms

| ***Key concepts*** | ***Health*** | ***Governance*** | ***Assess*** | ***Measuring tools*** | ***Global*** |
|---|---|---|---|---|---|
| ***Search terms*** | health | 1. governance<br>2. leadership<br>3. accountability<br>4. stewardship<br>5. transparency<br>6. policy development/formulation<br>7. strategic vision/direction<br>8. partnership<br>9. participation<br>10. involvement<br>11. consensus | 1. evaluate<br>2. monitor<br>3. measure<br>4. assess | 1. indicator<br>2. score<br>3. index | 1. global<br>2. international<br>3. world<br>4. multi-country |

The authors will search the following bibliographic databases: PubMed, Embase and Web of Science Core Collection. Appendix C in the supplemental material presents a full search strategy for each electronic database.

Given that some indices or assessment tools might not be commercially or academically published, grey literature will be an essential source of information in this scoping review. Google will be searched using a de-customised mode. Other search tools will include WHO Institutional Repository for Information Sharing (IRIS). In addition, experts in global health will be consulted to explore additional literature sources.

### Citation Tracking

As the meaning of "governance" in this scoping review might not be apparent in the existing indices or assessment tools, citation tracking will be used to identify relevant articles. One approach is backwards snowballing (reference searching) through reviews or literature citing a potentially eligible index or assessment tool. For example, the scoping review by Chiossi, Tsolova, and Ciotti might have included some potential eligible literature for this scoping review.(23) Another approach is forward snowballing (cited by searching) through eligible literature. Citation tracking in the related field of literature can support us in finding additional indices and assessment tools.


For peer review only - http://bmjopen.bmj.com/site/about/guidelines.xhtml

## Selection of eligible studies

The literature that meets all the inclusion criteria will be included, while literature that meets any one of the exclusion criteria will be excluded. **Table 2** presents the eligibility criteria, following the SOCT (Subjects, Objectives, Coverage, Type of sources) framework developed by the authors. Appendix D in the supplemental material presents detailed eligibility criteria to assist the reviewers' decision in study selection.

All literature searched through bibliographic databases will be uploaded to Covidence, which will identify and remove duplications. Based on the eligibility criteria, two independent reviewers will screen the titles and abstracts (and full texts if no clues are helping to judge the eligibility) and then assess the full texts in detail to select the literature. However, for Google and WHO IRIS, another two reviewers will de-customise the searching, export the results for each search string to Excel, screen the titles and abstracts, summaries, or introductions if applicable, and then assess the full texts in detail separately. Literature obtained from citation tracking will be selected after the selection process of literature obtained from electronic searches.

**Table 2**. Eligibility criteria: SOCT framework

| | ***Inclusion criteria*** | ***Exclusion criteria*** |
|---|---|---|
| *Subjects* | Indices or assessment tools on human, animal, and/or environmental health governance with measurable indicators | Assessment frameworks or conceptual frameworks, or narrative assessments without measurement; on topics irrelevant to health |
| *Objectives* | Describing the indices or assessment tools (including. indicators or scoring system) | Only criticising, mentioning, and analysing the indices or assessment tools while not aiming to yield assessment results for health governance |
| *Coverage* | Can be applied in multiple countries at the global level | Applied or can only be applied within one country, one region or one type of specific organisations or individuals (e.g., hospital, enterprise); only appearing as a case study without further generalisation |
| *Type of sources* | Reports, documents, peer-reviewed publications, websites | Commentaries, editorials, reviews, blogs, letters, conference abstracts, protocols |

A pilot test with randomly selected 50 samples will be conducted. The reviewers will meet to discuss discrepancies and modify the eligibility criteria and elaboration document. The screening will only start when 75% agreement is achieved.(25)

The reasons for any exclusion following the full-text review will be recorded. The reviewers will resolve disagreements through discussions throughout the selection process. A third reviewer will make the final decision if the two paired reviewers cannot resolve the disagreement.

The search results and the study selection process will be reported in the final scoping review and presented in a PRISMA extension for scoping review (PRISMA-ScR) flow diagram.(26) All data will be recorded and exported into Excel form after the whole process ends.

## Data extraction

Two reviewers will extract data from the eligible literature independently using a tailored data extraction tool developed by the authors (**Table 3**). If discrepancies occur during the data extraction process, the two reviewers will discuss to reach a common decision. If there is an unsolved disagreement, a third reviewer will make the final decision. There will be a pilot test to ensure consistency among the reviewers.


For peer review only - http://bmjopen.bmj.com/site/about/guidelines.xhtml

**Table 3**. Draft data extraction form

| ***Extraction category*** | ***Description*** | | ***Data type*** |
|---|---|---|---|
| *Name* | Full name of the index or assessment tool | | Unstructured text |
| *Developer* | Author or agency that developed the index or assessment tool | | Unstructured text |
| *Reference* | The reference information of the index or assessment tool | | Unstructured text |
| *Time coverage* | First publication year | | Numerical data |
| | Publication frequency | Number | Numerical data |
| | | Annual, biennial, quarterly, monthly, etc. | Categorical data |
| | The coverage of years the index or assessment tool being used | | Numerical data |
| *Operation, if applicable* | Roles and coordination among sponsor, funder, manager or other stakeholders. | | Unstructured text |
| *Domain* | Human health, animal health, environmental health, etc. | | Categorical data |
| *Issues to address* | The health issues to address, e.g., health system strengthening, health security or health data. | | Categorical data |
| *Objectives* | The purpose of index or assessment tool creation; the assessed subjects. | | Unstructured text |
| *Geographic coverage* | Number of countries assessed | | Numerical data |
| | The geographic regions of countries assessed, e.g., Asia, Africa, Europe, North America, South America or global. | | Categorical data |
| *Implementation level* | The implementation level that the index or assessment tool was designed to assess, e.g., global, transnational, regional, national, subnational or local level | | Categorical data |
| *Dimensions* | The indicator dimensions (not the specific indicators) of assessment content, e.g., leadership, accountability, transparency and policy development | | Categorical data |
| *Indicators* | The indicators measuring health governance | | Unstructured text |
| *Theory or logic, if applicable* | The theory or logic based to develop the index or assessment tool | | Unstructured text |
| *Methods of index or assessment tool development* | Methods of design and development of the index or assessment tool, e.g., Delphi, review of literature or modelling. | | Categorical data |
| *Methods of data collection* | The approach used to obtain information necessary for the assessment, e.g., questionnaire, checklist, interview or secondary data collection | | Categorical data |
| *Methods of yielding results* | Methods of yielding assessment results, e.g., qualitative, quantitative or mixed methods and the corresponding specific methods | | Categorical data |
| *Types of assessment results (if there are any open ones)* | Types of results present the assessment results, e.g., scores, rankings and ratings | | Categorical data |
| *Validity and reliability, if applicable* | Description of the validation process or reliability check of the assessment | | Unstructured text |

The authors might modify the draft data extraction form during data extraction. The scoping review will detail the modifications compared with this protocol.

## Data presentation and analysis

A qualitative content analysis will follow the data extraction form to explicate further and compare each index or assessment tool.

Tables and figures will present the extracted data for each extraction category, followed by detailed descriptive analyses. An overview table will show the basic information of each eligible literature, including the name, developer and references. Then, numerical or categorical data will be calculated on counts and proportions. For instance, there might be N (p%) articles using Delphi approaches to develop the indices and assessment tools. Such statistics will help grasp an overview of the characteristics of the eligible literature. For unstructured texts, a qualitative data analysis software will be used for coding. The contents related to governance will be particularly coded. However, the data of



For peer review only - http://bmjopen.bmj.com/site/about/guidelines.xhtml

some extraction categories with unstructured texts could probably transfer to numerical or categorical data. For example, theory or logic might be further categorised by disciplines.

To better identify the advantages and gaps and summarise the lessons learned, there will be an analysis based on the proposed preliminary evaluation framework (**Table 4**) after the data presentation. This framework is amended from Haeberer's framework(22) by accommodating the topic of this scoping review and cutting the contents relying on the authors' subjective judgement. The purpose of this framework is not to set criteria for the indices or assessment tools. Instead, it is simply to guide a further deep discussion based on the descriptive data.

**Table 4.** Preliminary evaluation framework

| ***Criteria*** | ***Description*** |
|---|---|
| *Indicator completeness* | The extent to which the indicator system is complete and operationalised in the following ways (including but not limited to):<br>(1) The indicators can be assigned a direct value without following implicit indicators or questions;<br>(2) The indicators are predefined and organised, not being example indicators. |
| *Clarity of measurement parameters* | The extent to which the methods for measurement of the indicators, actions, or structures are stated |
| *Being evidence-based* | The extent to which the observational or experimental evidence is provided for assigning value to the indicators |
| *Feasibility* | The extent to which the index or assessment tool could be applied in multi-country settings in the following ways (including but not limited to):<br>(1) It is inclusive of disparities of countries, with universal or flexible indicators and available data;<br>(2) A management structure or accountable entity has been or is to be set for the long-term operation of the index or assessment tool. |
| *Utility* | The extent to which the index or assessment tool supports decisions related to improvement (aiming at internal audiences) or accountability (aiming at external stakeholders), and policy advocacy or other functions. |
| *Sustainability* | The extent to which the index or assessment tool could be applied continuously in the following ways (including but not limited to):<br>(1) It has a long-term operating plan, or it has been applied for multiple years;<br>(2) It accommodates changes in the health issues or other conditions;<br>(3) It has predictable long-term technical, managerial and financing support for daily functioning. |

Following the analysis above, this scoping review will discuss the feasibility and necessity of developing a new global health governance index or consensus framework. The feasibility evaluation in Table 4 will facilitate the feasibility analysis at this stage, and the gaps identified above will assist the necessity analysis. Therefore, the study will inform future research and practices in assessing global health governance.

## Patient and public involvement

Patients and the public will not be involved in this scoping review.

# ETHICS AND DISSEMINATION

The analytical results will inform various stakeholders, including researchers, public health agencies, governments, global health organisations, and other health governance actors. Dissemination of this scoping review will include publication in a peer-reviewed scientific journal, policy briefs and conference presentations. Ethics approval is not required as the data are available publicly.


For peer review only - http://bmjopen.bmj.com/site/about/guidelines.xhtml

## Acknowledgements

The authors are grateful to two librarians, Muriel Leclerc from the University of Geneva and Yi Ren from Tsinghua University, for their guidance on the search strategy. Also, thank Marhaba Abdurëhim, Junpeng Wang, and Yunke Gu for doing the initial search to check the availability of the potential indices or assessment tools.


## Author Contributions

All authors contributed to the study's design, drafted the manuscript, provided feedback, and approved the final manuscript. KT provided feedback in principle, oversaw revisions and refined the manuscript. KT will also be the guarantor of the review. AH developed the search strategy, eligibility criteria, and data extraction tool and drafted and edited the protocol. YL structured the protocol, drafted the methods, refined the search strategy and eligibility criteria in detail, and contributed to the pilot tests. LZ drafted the introduction session and the supplemental material, refined the search strategy and the manuscript, and contributed to the pilot tests. JD developed the initial search strategy and eligibility criteria and drafted the methods. QH led the initial search to check the availability of the potential indices or assessment tools.


## Funding

This work is supported by the Research Fund of Vanke School of Public Health, Tsinghua University (No. 2021ZZ004) and Spring Breeze Fund, Tsinghua University (No. 20203080035). The funders are not involved in the research. They will also have no input on interpreting or publishing the study results.


## Competing interests

None declared.

## Patient consent for publication

None declared.

## Data statement

The data is not applicable as this article is only a protocol. The data will be accessed in an open repository for the scoping review. When requested, the authors will provide missing or additional data.

For peer review only

# REFERENCES

1. Chhotray V, Stoker G. Governance: From Theory to Practice. In: Chhotray V, Stoker G, editors. Governance Theory and Practice: A Cross-Disciplinary Approach [Internet]. London: Palgrave Macmillan UK; 2009 [cited 2022 Mar 23]. p. 214–47. Available from: https://doi.org/10.1057/9780230583344_10

2. Rhodes RAW. Understanding Governance: Ten Years On. Organ Stud. 2007 Aug;28(8):1243–64.

3. World Health Organization. Monitoring the building blocks of health systems: a handbook of indicators and their measurement strategies [Internet]. Geneva: World Health Organization; 2010 [cited 2022 Mar 23]. Available from: https://apps.who.int/iris/handle/10665/258734

4. Frenk J, Moon S. Governance Challenges in Global Health. N Engl J Med. 2013 Mar 7;368(10):936–42.

5. de Campos TC. Guiding Principles of Global Health Governance in Times of Pandemics: Solidarity, Subsidiarity, and Stewardship in COVID-19. Am J Bioeth. 2020 Jul 2;20(7):212–4.

6. Gostin LO, Moon S, Meier BM. Reimagining Global Health Governance in the Age of COVID-19. Am J Public Health. 2020 Oct 7;110(11):1615–9.

7. West-Oram PGN, Buyx A. Global Health Solidarity. Public Health Ethics. 2017 Jul 1;10(2):212–24.

8. Global Health 50/50. The global health 50/50 report 2020: power, privilege and priorities. London, UK; 2020.

9. Larson C. Global health indicators: an overview. Can Med Assoc J. 2004 Nov 9;171(10):1199–200.

10. Kirigia JM, Kirigia DG. The essence of governance in health development. Int Arch Med. 2011 Mar 28;4(1):11.

11. Peters MDJ, Marnie C, Tricco AC, Pollock D, Munn Z, Alexander L, et al. Updated methodological guidance for the conduct of scoping reviews. JBI Evid Implement. 2021 Mar;19(1):3–10.

12. Munn Z, Peters MDJ, Stern C, Tufanaru C, McArthur A, Aromataris E. Systematic review or scoping review? Guidance for authors when choosing between a systematic or scoping review approach. BMC Med Res Methodol. 2018 Nov 19;18(1):143.

13. Lee K, Brumme ZL. Operationalising the One Health approach: the global governance challenges. Health Policy Plan. 2013 Oct 1;28(7):778–85.

14. Mohammed K, Ahmed B. The governance of basic health coverage: A systematic review. Ethiop J Health Dev [Internet]. 2020 Oct 22 [cited 2021 Nov 20];34(3). Available from: https://www.ajol.info/index.php/ejhd/article/view/201342

15. Ntr A, M RA, C LP. The Literature Review of the Governance Frameworks in Health System. J Public Adm Gov. 2019 Sep 29;9(3):252.

16. Pyone T, Smith H, van den Broek N. Frameworks to assess health systems governance: a systematic review. Health Policy Plan. 2017 Jun 1;32(5):710–22.

17. Ravi SJ, Meyer D, Cameron E, Nalabandian M, Pervaiz B, Nuzzo JB. Establishing a theoretical foundation for measuring global health security: a scoping review. BMC Public Health. 2019 Jul 17;19(1):954.

For peer review only

18. Assefa Y, Nigusie A, Gedle D, Debie A. An integrated health system resilience framework to realise universal health coverage and health security: a systematic review. PROSPERO; 2020. Report No.: CRD42020210471.

19. Debie A, Khatri R, Assefa Y. Successes and challenges of health system governance towards universal health coverage and global health security: a systematic review [Internet]. PROSPERO; 2021 [cited 2021 Nov 18]. Report No.: CRD42021270090. Available from: https://www.crd.york.ac.uk/prospero/display_record.php?RecordID=270090

20. Mikkelsen-Lopez I, Wyss K, de Savigny D. An approach to addressing governance from a health system framework perspective. BMC Int Health Hum Rights. 2011 Dec 2;11(1):13.

21. Fehr R, Alexanderson K, Favaretti C, de Jong J, La Torre G, Lim TA, et al. Health assessments for health governance—concepts and methodologies. Eur J Public Health. 2017 Aug 1;27(4):609–16.

22. Haeberer M, Tsolova S, Riley P, Cano-Portero R, Rexroth U, Ciotti M, et al. Tools for Assessment of Country Preparedness for Public Health Emergencies: A Critical Review. Disaster Med Public Health Prep. 2021 Aug;15(4):431–41.

23. Chiossi S, Tsolova S, Ciotti M. Assessing public health emergency preparedness: A scoping review on recent tools and methods. Int J Disaster Risk Reduct. 2021 Apr 1;56:102104.

24. Arksey H, O'Malley L. Scoping studies: towards a methodological framework. Int J Soc Res Methodol. 2005 Feb;8(1):19–32.

25. Peters M, Godfrey C, McInerney P, Munn Z, Trico A, Khalil H. Chapter 11: Scoping Reviews. In: Aromataris E, Munn Z, editors. JBI Manual for Evidence Synthesis [Internet]. JBI; 2020 [cited 2021 Nov 11]. Available from: https://jbi-global-wiki.refined.site/space/MANUAL/3283910770/Chapter+11%3A+Scoping+reviews

26. Tricco AC, Lillie E, Zarin W, O'Brien KK, Colquhoun H, Levac D, et al. PRISMA Extension for Scoping Reviews (PRISMA-ScR): Checklist and Explanation. Ann Intern Med. 2018 Oct 2;169(7):467–73.

27. Page MJ, Moher D, Bossuyt PM, Boutron I, Hoffmann TC, Mulrow CD, et al. PRISMA 2020 explanation and elaboration: updated guidance and exemplars for reporting systematic reviews. BMJ. 2021 Mar 29;n160.

28. Rethlefsen ML, Kirtley S, Waffenschmidt S, Ayala AP, Moher D, Page MJ, et al. PRISMA-S: an extension to the PRISMA Statement for Reporting Literature Searches in Systematic Reviews. Syst Rev. 2021 Jan 26;10(1):39.

29. Shamseer L, Moher D, Clarke M, Ghersi D, Liberati A, Petticrew M, et al. Preferred reporting items for systematic review and meta-analysis protocols (PRISMA-P) 2015: elaboration and explanation. Res METHODS. 2015;25.

30. Moher D, Shamseer L, Clarke M, Ghersi D, Liberati A, Petticrew M, et al. Preferred reporting items for systematic review and meta-analysis protocols (PRISMA-P) 2015 statement. Syst Rev. 2015 Dec;4(1):1.

31. McGowan J, Sampson M, Salzwedel DM, Cogo E, Foerster V, Lefebvre C. PRESS Peer Review of Electronic Search Strategies: 2015 Guideline Statement. J Clin Epidemiol. 2016 Jul 1;75:40–6.

For peer review only

# Appendix A to Appendix D

## Appendix A. KEY CONCEPTS

### Governance

Governance has been studied in various dimensions, such as socio-legal studies, political science, economics, and development studies. In a broader term, governance is described as a series of collective actions and decision-making procedures with diverse actors and organisations without formal control mechanisms. Governance does not depend on authority and coercion; it is achieved through negotiation, communication, and hegemonic influence.(1) It does not only concern the government, and it emphasises governing with and through networks between public, private and voluntary sectors.(2) The United Nations Development Programme (UNDP) refers to good governance broadly as the principles of legitimacy and voice, direction, performance, accountability, and fairness.(3) Definition of governance World Health Organization (WHO) focuses on effective oversight, coalition-building, the provision of regulations and incentives, attention to system-design and accountability.(4) Concerning health, Baez-Camargo and Jacobs define governance as “processes through which health systems manage human resources, acquire and distribute medicines and technologies, generate and disseminate information, and provide means to finance the provision of health services to the population.” (5)

Given the inclusiveness of “governance”, this scoping review does not aim to define a concrete *priori* concept of governance. Rather, it tried extensively potential search terms according to the existing analysis of governance dimensions (**Table A**) through a rapid review of reviews (systematic review, scoping review or systematic-like reviews).[1] Three reviews were eligible as they synthesised the literature surrounding the concept of “governance”. The first column lists the elements, dimensions or functions (referred to as “dimensions) of “governance” identified by these three reviews or their search terms for “governance”. To further identify the more recognised key dimensions of “governance”, this column only shows the dimensions or search terms overlapped by at least two reviews. Terms with similar meanings are classified in one group, such as participation and involvement. The second to fourth column shows the original expressions of the classified dimensions or search terms in the corresponding reviews.

Based on Table A, we develop the search terms for the concept “governance” in our search strategy. The search terms cover all the dimensions or search terms for “governance” overlapped in the included review listed in Table A.

Besides, we also referred to search strategies of existing systematic reviews or scoping reviews on health governance[2].(6–12) Apart from “governance” itself and the dimensions listed above, we add

[1] We used the search strings “governance AND (concepts OR concept OR definition* OR define OR defining OR meaning*)” in Web of Science, Ovid (Embase <1974 to 2022 March 11> and Medline and Epub Ahead of Print, In-Process, In-Data-Review & Other Non-Indexed Citations, Daily and Versions <1946 to March 11, 2022>), Cochrane, and Google Scholar. The search field was “Title” in bibliographic databases, the time frame was 2000-2021 and the document type was review article. Without removing duplicates, the searching results are 360, 178 and 5220 respectively in Web of Science, Ovid and Cochrane. In order to find additional literature to the bibliographic databases, there is no search field in Google Scholar and the top 100 items by relevance were screened. The search date was 14 March 2022. In addition, in Barbazza et al. (2014), broad dimensions include some fundamental values and outcomes that could be independent from the concept of governance, so we only list the functional dimensions according to this article in Table A.

[2] We used the search strings “health NEAR/5 governance’ AND (‘systematic review’ OR ‘scoping review’)” in Web of Science and Ovid (Embase <1974 to 2022 March 11> and Medline and Epub Ahead of Print, In-Process, In-Data-Review & Other Non-Indexed Citations, Daily and Versions <1946 to March 11, 2022>). The search field was “Title” and the time frame was 2000-2021. Without removing duplicates, the searching results are 15 and 10 respectively in Web of Science and Ovid. The search date was 14 March 2022. The inclusion criteria are: (1) using governance as a concept to search; (2) the search terms in the governance concept are more than “governance” itself; (3) the topic is health related governance, not data or other governance or other governance

another term, "leadership", for two reasons. First, it has been widely used as a search term for the concept of "governance". We did another review of reviews focusing on search terms used in systematic or scoping reviews on health governance and found that three out of seven eligible reviews used "leadership" as a search term for the concept of "governance".(6–8) Except for "governance" per se, only "accountability" appears in these reviews more than "leadership" does (four times). Second, leadership entails "the capacity of the system to initiate, implement and monitor a regulatory system" and "the ability to organise and manage the alignment of all relevant actors and actions engaged in processes pertaining to this".(13) Thus, it is often linked to governance, as explicated in governance literature (13,14) and potentially eligible literature in this scoping review like Leadership and Governance for Health Indicators.(15)

**Table A.** Summary of reviews regarding governance dimensions

| *Information of eligible reviews* / *Classified dimensions or search terms for "governance"* | *Carlson et al., "Defining the functions of public health governance." American Journal of Public Health (2015).(14)* | *Barbazza and Tello, "A review of health governance: definitions, dimensions and tools to govern." Health Policy (2014). (13)* | *Ruhanen et al. "Governance: a review and synthesis of the literature." Tourism Review (2010).(16)* |
|---|---|---|---|
| *Accountability* | - | Accountability | Accountability |
| *Stewardship* | Resource stewardship | Stewardship | - |
| *Transparency* | - | Transparency | Transparency |
| *Policy development/formulation* | Policy development | Formulating policy/strategic direction | - |
| *Strategic vision/direction* | - | Formulating policy/strategic direction | Strategic vision |
| *Partner/partnership* | Partner engagement | Partnerships | - |
| *Participation/involvement* | - | Participation and consensus | Involvement |
| *Consensus* | - | Participation and consensus | Consensus |

## Global Health Governance

While titled "health governance across countries", this scoping review is embedded in the context of global health governance. Although existing literature has centred on governance in health systems and health development only in recent decades, there has been an increasing interest in discussing the relationship between governance and global health. Despite the extensive scholarly debates on the definition of global health, it is still elusive to reach a consensus around a precise definition. In 2009, Koplan and colleagues argued for "a common definition of global health", which emphasises transnational health, and embraces different disciplines and interdisciplinary collaboration.(17) Scholars delineate global health by focusing on multiple dimensions, such as education, governance and security. Many also view global health as a mode of governance across borders.(18) The term global health governance (GHG) is widely used in scholarly work, but few researchers agree on how the term should be applied. Lee and Kamradt-Scott point out that the GHG peer-reviewed literature varies substantially on what kinds of and to what extent institutions should engage in GHG. In addition, the goal and function of GHG are also not clear.(19) For instance, Kickbusch and Szabo refer GHG mainly to institutions and governance processes that are directly linked to health, such as the WHO.(20) Another highly cited definition proposed by David P. Fidler takes a more inclusive approach. He defines GHG as "the use of formal and informal institutions, rules, and processes by states,

linking to health; (4) systematic review or scoping review. Seven literature is eligible as a result of this review of reviews.

intergovernmental organisations, and non-state actors to deal with challenges to health that require cross-border collective action to address effectively." (21)

# Appendix B. PRELIMINARY SEARCHES

The methods for searching related published/ongoing reviews were: (1) directly searching using strings "'health governance' AND ('index' OR 'indicator' OR assess) AND ('systematic review' OR 'scoping review')"; (2) forward snowballing for the reviews including our potential eligible literature such as GHSI and IHR core capacity, using strings "('global health security index' OR 'GHSI') AND ('systematic review' OR 'scoping review')" and "('international health regulations' OR 'IHR') AND ('systematic review' OR 'scoping review') AND ('core capacity' OR 'score')". There was no filter or limit, and the search dates were 20 November 2021 and 22 March 2022.

# Appendix C. FULL SEARCH STRATEGY

## Web of science Core Collection

Search date: 03 April 2022

| *No.* | *Query* | *Results* |
|---|---|---|
| #1 | TS=("health" OR "healths" OR "healthcare*") | 2,899,246 |
| #2 | TS=("governance*" OR "leadership*" OR "accountabilit*" OR "stewardship*" OR (("policy" OR "policies") NEAR/10 ("develop" OR "developing" OR "develops" OR "developed" OR "development" OR "developments" OR "formulat*")) OR ("strateg*" NEAR/10 ("vision" OR "visions" OR "direction" OR "directions")) OR "partner*" OR "transparen*" OR "participation*" OR "involvement*" OR "consensus*") | 2,178,485 |
| #3 | TS=("evaluate" OR "evaluated" OR "evaluates" OR "evaluating" OR "evaluation*" OR "monitor" OR "monitors" OR "monitored" OR "monitoring" OR "measure " OR "measures" OR "measured" OR "measuring" OR "measurement*" OR "assess" OR "assesses" OR "assessed" OR "assessing" OR "assessment*") | 15,885,119 |
| #4 | TS=("indicator*" OR "score" OR "scores" OR "scored" OR "scoring" OR "index" OR "indexs" OR "indexes" OR "indexed" OR "indices" OR "indexing") | 3,259,711 |
| #5 | TS=("globe" OR "global" OR "globally" OR "international" OR "internationally" OR "world" OR "worldwide" OR "worldwidely" OR "multi country " OR "multi countries") | 3,745,205 |
| #6 | #1 AND #2 AND #3 AND #4 AND #5 AND DOP=(2000-01-01/2021-12-31) | 8,046 |
| #7 | #7 NOT DT=(Review OR Editorial Material OR Meeting Abstract OR Letter) | 6,844 |

## PubMed

Search date: 03 April 2022

| *No* | *Query* | *Results* |
|---|---|---|
| #1 | "health"[MeSH Terms] OR "health"[Title/Abstract] OR "healths"[Title/Abstract] OR "healthcare*"[Title/Abstract] | 2,617,637 |
| #2 | "leadership"[MeSH Terms] OR "public policy"[MeSH Terms] OR "consensus"[MeSH Terms] OR "social participation"[MeSH Terms] OR "community participation"[MeSH Terms] OR "stakeholder participation"[MeSH Terms] OR "governance*"[Title/Abstract] OR "leadership*"[Title/Abstract] OR "accountabilit*"[Title/Abstract] OR "stewardship*"[Title/Abstract] OR (("policy"[Title/Abstract] OR "policies"[Title/Abstract]) AND (("develop"[Title/Abstract] OR "developing"[Title/Abstract] OR "develops"[Title/Abstract] OR "developed"[Title/Abstract] OR "development"[Title/Abstract] OR "developments"[Title/Abstract]) OR "formulat*"[Title/Abstract])) OR ("strateg*"[Title/Abstract] AND ("vision"[Title/Abstract] OR "visions"[Title/Abstract] OR "direction"[Title/Abstract] OR "directions"[Title/Abstract])) OR "partner*"[Title/Abstract] OR "transparen*"[Title/Abstract] OR "participation*"[Title/Abstract] OR | 1,434,193 |


For peer review only - http://bmjopen.bmj.com/site/about/guidelines.xhtml

| | | |
|---|---|---|
| | "involvement*"[Title/Abstract] OR "consensus*"[Title/Abstract] | |
| #3 | "evaluate"[Title/Abstract] OR "evaluated"[Title/Abstract] OR "evaluates"[Title/Abstract] OR "evaluating"[Title/Abstract] OR "evaluation*"[Title/Abstract] OR "monitor"[Title/Abstract] OR "monitors"[Title/Abstract] OR "monitored"[Title/Abstract] OR "monitoring"[Title/Abstract] OR "measure"[Title/Abstract] OR "measures"[Title/Abstract] OR "measured"[Title/Abstract] OR "measuring"[Title/Abstract] OR "measurement*"[Title/Abstract] OR "assess"[Title/Abstract] OR "assesses"[Title/Abstract] OR "assessed"[Title/Abstract] OR "assessing"[Title/Abstract] OR "assessment*"[Title/Abstract] | 9,147,656 |
| #4 | "indicator*"[Title/Abstract] OR "score"[Title/Abstract] OR "scores"[Title/Abstract] OR "scored"[Title/Abstract] OR "scoring"[Title/Abstract] OR "index"[Title/Abstract] OR "indexs"[Title/Abstract] OR "indexes"[Title/Abstract] OR "indexed"[Title/Abstract] OR "indices"[Title/Abstract] OR "indexing"[Title/Abstract] | 2,311,393 |
| #5 | "globe"[Title/Abstract] OR "global"[Title/Abstract] OR "globally"[Title/Abstract] OR "international"[Title/Abstract] OR "internationally"[Title/Abstract] OR "world"[Title/Abstract] OR "worldwide"[Title/Abstract] OR "worldwidely"[Title/Abstract] OR "multi country"[Title/Abstract] OR "multi countries"[Title/Abstract] | 1,490,795 |
| #6 | #1 AND #2 AND #3 AND #4 AND #5 | 7,928 |
| #7 | #1 AND #2 AND #3 AND #4 AND #5 AND ("2000/01/01"[Date - Publication] : "2021/12/31"[Date - Publication]) | 7,407 |
| #8 | (#7) NOT (Comment[Publication Type] OR Editorial[Publication Type] OR Letter[Publication Type] OR Review[Publication Type]) | 6,270 |

## Embase

Search date: 03 April 2022

| *No.* | *Query* | *Results* |
|---|---|---|
| #1 | 'health'/exp OR 'public health'/exp OR 'health care'/exp OR health:ab,ti,kw OR healths:ab,ti,kw OR 'healthcare*':ab,ti,kw | 11,573,270 |
| #2 | 'leadership'/exp OR 'policy'/exp OR 'consensus'/exp OR 'governance'/exp OR 'accountability'/exp OR 'strategy'/exp OR 'partner'/exp OR 'partnership'/exp OR 'participation'/exp OR 'involvement'/exp OR 'transparency'/exp OR 'governance*':ab,ti,kw OR 'leadership*':ab,ti,kw OR 'accountabilit*':ab,ti,kw OR 'stewardship*':ab,ti,kw OR (('policy' OR 'policies') NEAR/10 ('develop' OR 'developing' OR 'develops' OR 'developed' OR 'development' OR 'developments' OR 'formulat*')):ab,ti,kw OR ('strateg*' NEAR/10 ('vision' OR 'visions' OR 'direction' OR 'directions')):ab,ti,kw OR 'partner*':ab,ti,kw OR 'transparen*':ab,ti,kw OR 'participation*':ab,ti,kw OR 'involvement*':ab,ti,kw OR 'consensus*':ab,ti,kw | 1,866,006 |
| #3 | 'monitor'/de OR 'measurement'/de OR 'assessment'/exp OR evaluate:ab,ti,kw OR evaluated:ab,ti,kw OR evaluates:ab,ti,kw OR evaluating:ab,ti,kw OR evaluation*:ab,ti,kw OR monitor:ab,ti,kw OR monitors:ab,ti,kw OR monitored:ab,ti,kw OR monitoring:ab,ti,kw OR measure:ab,ti,kw OR measures:ab,ti,kw OR measured:ab,ti,kw OR measuring:ab,ti,kw OR measurement*:ab,ti,kw OR assess:ab,ti,kw OR assesses:ab,ti,kw OR assessed:ab,ti,kw OR assessing:ab,ti,kw OR assessment*:ab,ti,kw | 12,334,482 |
| #4 | 'indicator'/de OR 'score'/exp OR 'index'/exp OR indicator*:ab,ti,kw OR score:ab,ti,kw OR scores:ab,ti,kw OR scored:ab,ti,kw OR scoring:ab,ti,kw OR index:ab,ti,kw OR indexs:ab,ti,kw OR indexes:ab,ti,kw OR indexed:ab,ti,kw OR indices:ab,ti,kw OR indexing:ab,ti,kw | 3,358,824 |
| #5 | 'global'/exp OR 'world'/exp OR globe:ab,ti,kw OR global:ab,ti,kw OR globally:ab,ti,kw OR international:ab,ti,kw OR internationally:ab,ti,kw OR world:ab,ti,kw OR worldwide:ab,ti,kw OR worldwidely:ab,ti,kw OR 'multi country':ab,ti,kw OR 'multi countries':ab,ti,kw | 2,099,845 |
| #6 | #1 AND #2 AND #3 AND #4 AND #5 | 19,014 |
| #7 | #1 AND #2 AND #3 AND #4 AND #5 AND [2000-2021]/py | 18,199 |
| #8 | #7 NOT (editorial:it OR letter:it OR review:it OR 'conference abstract':it) | 9,416 |



For peer review only - http://bmjopen.bmj.com/site/about/guidelines.xhtml

## Google

Search date: 03 April 2022

Customed range: 2000-2021

| *No.* | *Query (Google limits queries to 32 words)* | *Results* |
|---|---|---|
| *1* | health AND (governance OR leadership OR accountability OR stewardship) AND (evaluate OR monitor OR measure OR assess) AND (index OR indicator OR score) AND (global OR international OR world OR multi-country) | 151 |
| *2* | health AND (transparency OR policy OR strategy) AND (evaluate OR monitor OR measure OR assess) AND (index OR indicator OR score) AND (global OR international OR world OR multi-country) | 178 |
| *3* | health AND (partnership OR participation OR involvement OR consensus) AND (evaluate OR monitor OR measure OR assess) AND (index OR indicator OR score) AND (global OR international OR world OR multi-country) | 157 |

# Appendix D. ELIGIBILITY CRITERIA IN DETAIL

| | *Inclusion criteria* | *Exclusion criteria* |
|---|---|---|
| *Subjects* | Indices or assessment tools on human, animal, and/or environmental health governance with measurable indicators:<br>1.1 General health governance;<br>1.2 Governance of health system;<br>1.3 Governance of public health risks such as public health emergencies, epidemics and pandemics;<br>1.4 Governance on specific health issues such as influenza, reproductive health. | Assessment/conceptual frameworks, or narrative assessment without measurement; on topics irrelevant to health<br>1.1 Not being an index or assessment tool;<br>1.2 Irrelevant to our topics<br>1.2.1 Not governance-related;<br>1.2.2 Not health-related;<br>1.2.3 Health outcome-based;<br>1.2.4 Both non-governance- and non-health-related |
| *Objectives* | 2 Describing the indices or assessment tools (incl. indicators or scoring results) | 2 Only criticising, mentioning, and analysing the indices or assessment tools while not aiming to yield assessment results for health governance |
| *Coverage* | 3 Can be applied in multi-countries at the global level (although the assessed subject might be sub-national/national entities) | 3.1 Geographical coverage: Only applied/can be applied within one country or one region;<br>3.2 Entity coverage: Assessing one type of specific organisations or individuals (e.g., hospital or enterprise)<br>3.3 Case study: Appearing as a case study without further generalisation |
| *Type of sources* | 4 Reports, documents, peer-reviewed publications, websites | 4 Wrong publication types: commentaries, reviews, blogs, protocols |

## REFERENCES

1. Chhotray V, Stoker G. Governance: From Theory to Practice. In: Chhotray V, Stoker G, editors. Governance Theory and Practice: A Cross-Disciplinary Approach [Internet]. London: Palgrave Macmillan UK; 2009 [cited 2022 Mar 23]. p. 214–47. Available from: https://doi.org/10.1057/9780230583344_10

2. Rhodes RAW. Understanding Governance: Ten Years On. Organ Stud. 2007 Aug;28(8):1243–64.

3. Nations DPU. Governance for sustainable human development: A UNDP policy document. UNDP; 1997.

4. World Health Organization. Everybody's business–strengthening health systems to improve health outcomes: WHO's framework for action. 2007;

5. Baez Camargo C, Jacobs E. A framework to assess governance of health systems in low income countries. 2011;

6. Hastings S, Armitage G, Mallinson S, Jackson K, Suter E. Exploring the relationship between governance mechanisms in healthcare and health workforce outcomes: a systematic review. BMC Health Serv Res. 2014 Oct 4;14.

7. Pyone T, Smith H, van den Broek N. Frameworks to assess health systems governance: a systematic review. Health Policy Plan. 2017 Jun 1;32(5):710–22.

8. Abd Rahim A, Manaf R, Juni M, Ibrahim N. Health System Governance for the Integration of Mental Health Services into Primary Health Care in the Sub-Saharan Africa and South Asia Region: A Systematic Review. Inq- J Health CARE Organ Provis Financ. 2021 Jul;58.

9. Mohammed K, Ahmed B. The governance of basic health coverage: A systematic review. Ethiop J Health Dev [Internet]. 2020 Oct 22 [cited 2021 Nov 20];34(3). Available from: https://www.ajol.info/index.php/ejhd/article/view/201342

10. Naher N, Hoque R, Hassan M, Balabanova D, Adams A, Ahmed S. The influence of corruption and governance in the delivery of frontline health care services in the public sector: a scoping review of current and future prospects in low and middle-income countries of south and south-east Asia. BMC PUBLIC Health. 2020 Jun 8;20(1).

11. Ayanore M, Amuna N, Aviisah M, Awolu A, Kipo-Sunyehzi D, Mogre V, et al. Towards Resilient Health Systems in Sub-Saharan Africa: A Systematic Review of the English Language Literature on Health Workforce, Surveillance, and Health Governance Issues for Health Systems Strengthening. Ann Glob Health. 2019;85(1).

12. Tabrizi J.S., Pourasghar F., Nikjoo R.G. Primary health care governance pillars and components in developed countries, a systematic review. Int J Pharm Technol. 2016;8(4):24045–59.

13. Barbazza E, Tello JE. A review of health governance: Definitions, dimensions and tools to govern. Health Policy. 2014 May 1;116(1):1–11.

14. Carlson V, Chilton MJ, Corso LC, Beitsch LM. Defining the Functions of Public Health Governance. Am J Public Health. 2015 Apr;105(S2):S159–66.

15. Abt Associates. Leadership and Governance for Health Indicators | HFG [Internet]. The Health Finance and Governance (HFG) Project. [cited 2021 Nov 19]. Available from: https://www.hfgproject.org/health-governance-indicators/

16. Ruhanen L, Scott N, Ritchie B, Tkaczynski A. Governance: a review and synthesis of the literature. Pechlaner H, Raich F, Beritelli P, editors. Tour Rev. 2010 Jan 1;65(4):4–16.


For peer review only - http://bmjopen.bmj.com/site/about/guidelines.xhtml

17. Koplan JP, Bond TC, Merson MH, Reddy KS, Rodriguez MH, Sewankambo NK, et al. Towards a common definition of global health. The Lancet. 2009 Jun 6;373(9679):1993–5.

18. Salm M, Ali M, Minihane M, Conrad P. Defining global health: findings from a systematic review and thematic analysis of the literature. BMJ Glob Health. 2021;6:1–14.

19. Lee K, Kamradt-Scott A. The multiple meanings of global health governance: a call for conceptual clarity. Glob Health. 2014;10(1):28.

20. Kickbusch I, Szabo MMC. A new governance space for health. Glob Health Action. 2014 Feb 13;7(s2):23507.

21. Kickbusch I. Global Health Governance Challenges 2016 – Are We Ready? Int J Health Policy Manag. 2016 Feb 29;5(6):349–53.

For peer review only

# Reporting checklist for protocol of a systematic review and meta analysis.

Based on the PRISMA-P guidelines.

## Instructions to authors

Complete this checklist by entering the page numbers from your manuscript where readers will find each of the items listed below.

Your article may not currently address all the items on the checklist. Please modify your text to include the missing information. If you are certain that an item does not apply, please write "n/a" and provide a short explanation.

Upload your completed checklist as an extra file when you submit to a journal.

In your methods section, say that you used the PRISMA-Preporting guidelines, and cite them as:

Moher D, Shamseer L, Clarke M, Ghersi D, Liberati A, Petticrew M, Shekelle P, Stewart LA. Preferred Reporting Items for Systematic Review and Meta-Analysis Protocols (PRISMA-P) 2015 statement. Syst Rev. 2015;4(1):1.

| | | Reporting Item | Page Number |
|---|---|---|---|
| **Title** | | | |
| Identification | #1a | Identify the report as a protocol of a systematic review | 2 |
| Update | #1b | If the protocol is for an update of a previous systematic review, identify as such | N/A. Not an update. |
| **Registration** | | | |
| | #2 | If registered, provide the name of the registry (such as PROSPERO) and registration number | 5 |
| **Authors** | | | |
| Contact | #3a | Provide name, institutional affiliation, e-mail address of all protocol authors; provide physical mailing address of corresponding author | 2 |
| Contribution | #3b | Describe contributions of protocol authors and | 10 |

For peer review only

| | | | |
|---|---|---|---|
| | | identify the guarantor of the review | |
| **Amendments** | | | |
| | #4 | If the protocol represents an amendment of a previously completed or published protocol, identify as such and list changes; otherwise, state plan for documenting important protocol amendments | 5 |
| **Support** | | | |
| Sources | #5a | Indicate sources of financial or other support for the review | 10 |
| Sponsor | #5b | Provide name for the review funder and / or sponsor | 10 |
| Role of sponsor or funder | #5c | Describe roles of funder(s), sponsor(s), and / or institution(s), if any, in developing the protocol | 10 |
| **Introduction** | | | |
| Rationale | #6 | Describe the rationale for the review in the context of what is already known | 4 |
| Objectives | #7 | Provide an explicit statement of the question(s) the review will address with reference to participants, interventions, comparators, and outcomes (PICO) | 4 |
| **Methods** | | | |
| Eligibility criteria | #8 | Specify the study characteristics (such as PICO, study design, setting, time frame) and report characteristics (such as years considered, language, publication status) to be used as criteria for eligibility for the review | 4-5, 7 |
| Information sources | #9 | Describe all intended information sources (such as electronic databases, contact with study authors, trial registers or other grey literature sources) with planned dates of coverage | 6 |
| Search strategy | #10 | Present draft of search strategy to be used for at least one electronic database, including planned limits, such that it could be repeated | 6 |
| Study records - | #11a | Describe the mechanism(s) that will be used to | 7-8 |

For peer review only

| | | | |
|---|---|---|---|
| data management | | manage records and data throughout the review | |
| Study records - selection process | #11b | State the process that will be used for selecting studies (such as two independent reviewers) through each phase of the review (that is, screening, eligibility and inclusion in meta-analysis) | 7 |
| Study records - data collection process | #11c | Describe planned method of extracting data from reports (such as piloting forms, done independently, in duplicate), any processes for obtaining and confirming data from investigators | 7-8 |
| Data items | #12 | List and define all variables for which data will be sought (such as PICO items, funding sources), any pre-planned data assumptions and simplifications | 8 |
| Outcomes and prioritization | #13 | List and define all outcomes for which data will be sought, including prioritization of main and additional outcomes, with rationale | 8-9 |
| Risk of bias in individual studies | #14 | Describe anticipated methods for assessing risk of bias of individual studies, including whether this will be done at the outcome or study level, or both; state how this information will be used in data synthesis | N/A. Not a systematic review protocol. |
| Data synthesis | #15a | Describe criteria under which study data will be quantitatively synthesised | N/A. Not a quantitative scoping review protocol. |
| Data synthesis | #15b | If data are appropriate for quantitative synthesis, describe planned summary measures, methods of handling data and methods of combining data from studies, including any planned exploration of consistency (such as I2, Kendall's τ) | N/A. Not a quantitative scoping review protocol. |
| Data synthesis | #15c | Describe any proposed additional analyses (such as sensitivity or subgroup analyses, meta-regression) | N/A. Not a quantitative scoping review protocol. |
| Data synthesis | #15d | If quantitative synthesis is not appropriate, describe the type of summary planned | 8-9 |

For peer review only

| | | | |
|---|---|---|---|
| Meta-bias(es) | #16 | Specify any planned assessment of meta-bias(es) (such as publication bias across studies, selective reporting within studies) | N/A. Not a systematic review protocol. |
| Confidence in cumulative evidence | #17 | Describe how the strength of the body of evidence will be assessed (such as GRADE) | N/A. Not a systematic review protocol. |

For peer review only
